# PJA1 mediates the effects of astrocytic GPR30 on learning and memory in female mice

**DOI:** 10.1172/JCI165812

**Published:** 2023-09-15

**Authors:** Xinshang Wang, Yongli Jiang, Ban Feng, Xue Ma, Kun Zhang, Fan Yang, Zhenguo Liu, Le Yang, Jiao Yue, Liang Lu, Dake Song, Qingjuan Guo, Jingyu Qi, Xubo Li, Min Wang, Huinan Zhang, Jing Huang, Minggao Zhao, Shuibing Liu

**Affiliations:** 1 Department of Pharmacology, School of Pharmacy and; 2 Precision Pharmacy & Drug Development Center, Department of Pharmacy, Tangdu Hospital, Fourth Military Medical University, Xi’an, China.; 3 Department of Pharmacy, Northwest Women’s and Children’s Hospital, Xi’an, China.; 4 Department of Health Management, Tangdu Hospital, Fourth Military Medical University, Xi’an, China.

**Keywords:** Inflammation, Neuroscience, Memory, Pharmacology

## Abstract

Hormone replacement therapy (HRT) is not recommended for treating learning and memory decline in menopausal women because it exerts adverse effects by activating classic estrogen receptors ERα and ERβ. The membrane estrogen receptor G protein-coupled receptor 30 (GPR30) has been reported to be involved in memory modulation; however, the underlying mechanisms are poorly understood. Here, we found that GPR30 deletion in astrocytes, but not in neurons, impaired learning and memory in female mice. Astrocytic GPR30 depletion induced A1 phenotype transition, impairing neuronal function. Further exploration revealed that Praja1 (PJA1), a RING ubiquitin ligase, mediated the effects of astrocytic GPR30 on learning and memory by binding to Serpina3n, which is a molecular marker of neuroinflammation in astrocytes. GPR30 positively modulated PJA1 expression through the CREB signaling pathway in cultured murine and human astrocytes. Additionally, the mRNA levels of *GPR30* and *PJA1* were reduced in exosomes isolated from postmenopausal women while Serpina3n levels were increased in the plasma. Together, our findings suggest a key role for astrocytic GPR30 in the learning and memory abilities of female mice and identify GPR30/PJA1/Serpina3n as potential therapeutic targets for learning and memory loss in peri- and postmenopausal women.

## Graphical abstract

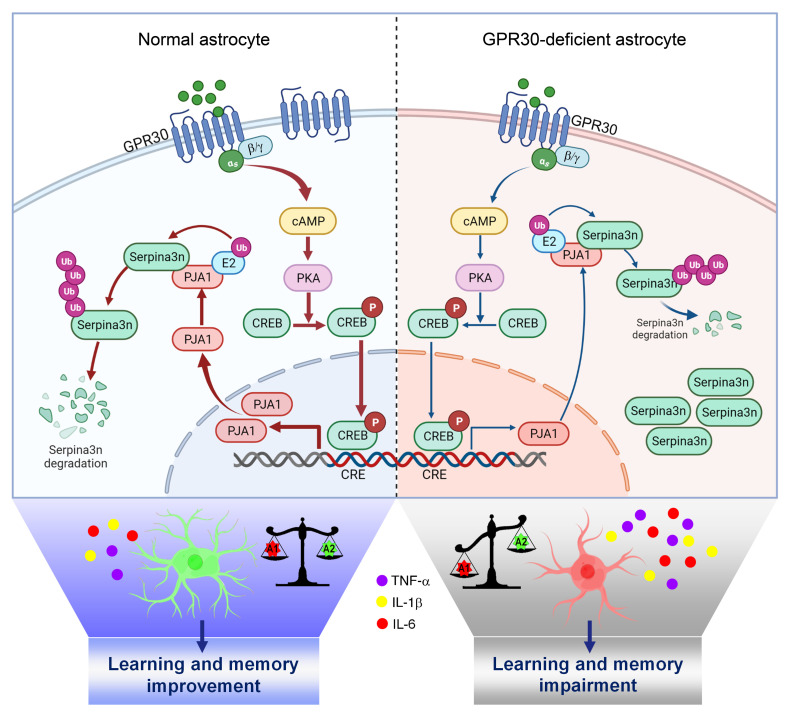

## Introduction

The process of learning and memory is how people acquire, encode, store, retain and later retrieve information in the brain. For perimenopausal women, learning and memory abilities decline rapidly due to a decrease in estrogen levels (1–3). Hormone replacement therapy (HRT) is an effective therapeutic strategy for ovariectomized (OVX) animals and menopausal women (4). Classic estrogen receptors — ERα and ERβ — and a specific membrane receptor, G protein-coupled receptor 30 (GPR30), also known as G protein-coupled estrogen receptor 1 (GPER1) mediate the effects of estradiol on cognition (5, 6). However, it is well known that long-term HRT increases the risks of cardiovascular disease, thromboembolic disease, breast cancer, and endometrial cancer (7). ERα and ERβ contribute to the progression of several hormone-responsive cancer types (8), especially breast cancer. Therefore, some researchers have begun to focus on the effectiveness of targeting other receptors, including GPR30. Several papers have reported that systemic activation of GPR30 can enhance spatial memory and social cognition (9, 10), but the mechanism remains largely unknown.

Emerging evidence suggests that astrocytes, the most abundant glial cells in the brain, are closely related to synaptic transmission and plasticity, contributing to learning and memory processes (11, 12). Recent studies have revealed that manipulating calcium (Ca^2+^) signaling in astrocytes by chemogenetic and optogenetic tools can directly modulate neuronal activity and memory acquisition and consolidation (13, 14). In addition, the physiological activities of astrocytes depend on their complex morphology; astrocytes are composed of an elaborate network of branches and processes that allow them to contact surrounding neuronal somata, dendrites, spines, and presynaptic terminals (15, 16). However, after brain injury and in brain diseases, astrocytes can be transformed to active cells called “reactive astrocytes” and undergo morphological changes. The functions of reactive astrocytes have been disputed, as they both hinder and support brain recovery (17, 18). Barres et al. purified and genetically profiled reactive astrocytes from 2 injury mouse models, namely neuroinflammation and ischemic stroke models (19). They found that neuroinflammation and ischemia induced the polarization of astrocytes toward 2 different phenotypes termed “A1” and “A2”, which are analogous to the “M1” and “M2” microglial phenotypes (20). The genes that are upregulated in A1 astrocytes destroy synapses, so A1 astrocytes are considered harmful. In contrast, the genes that are upregulated in A2 astrocytes include many neurotrophic factors that are protective. Serine peptidase inhibitor clade A member 3N (Serpina3n), a serine protease inhibitor of the serpin family, is mainly produced by reactive astrocytes from mouse models and patients (19, 21). It is recognized as a key marker of neuroinflammation in astrocytes (21).

Astrocyte dysfunction is closely associated with ubiquitination, which not only participates in protein degradation but also regulates protein activity by posttranslational modification (22, 23). Alterations in ubiquitination can result in structural abnormalities and/or the accumulation of certain proteins in cells and tissues, giving rise to numerous diseases, including neurodegenerative disorders (24, 25). Ubiquitination involves the sequential action of 3 enzymes. E1, a ubiquitin-activating enzyme, catalyzes the ATP-dependent activation of ubiquitin. Then, ubiquitin is transferred to E2 (ubiquitin-conjugating enzyme) and through E3 (ubiquitin ligase) to the substrate. E3s, the most heterogeneous class of enzymes in the ubiquitination pathway, can recognize the protein target (substrate) and catalyze the addition of ubiquitin by E2s. E3s are classified into 3 main types based on their characteristic domains and catalytic mechanisms: (a) really interesting new gene (RING), (b) homologous to E6AP C-terminus (HECT), and (c) RING-between-RING (RBR) E3s (26). RING E3s contain a zinc-binding domain or a U-box domain and mediate the direct transfer of ubiquitin to the substrate. Praja1 (PJA1), a RING E3, is highly expressed in the brain. The PJA1 gene is located in a specific region of the X-chromosome (27), and is related to neurodevelopmental disorders and is associated with craniofacial abnormalities and/or epilepsy (28). Moreover, PJA1 gene expression is downregulated in the amygdala in Alzheimer’s disease model mice and upregulated in the basolateral amygdala in mice during the formation of fear memory (29, 30), suggesting a potential role for PJA1 in learning and memory. How PJA1 is regulated and participates in the regulation of learning and memory is of interest.

In this study, we found, first, that selective deletion of GPR30 in astrocytes resulted in abnormal astrocyte function, which impaired learning and memory in female mice. Second, PJA1 was found to mediate the effects of astrocytic GPR30 on learning and memory by binding to Serpina3n. Last, the molecular mechanisms by which GPR30 regulates learning and memory were confirmed in samples from human female astrocytes and blood. These findings regarding the mechanisms by which GPR30 is involved in cognition will help us identify new strategies to treat learning and memory deficits in perimenopausal and postmenopausal women.

## Results

### GPR30 activation alleviated memory deficits in OVX mice.

Female 8-week-old mice were subjected to ovariectomy to mimic the ovarian function loss experienced by menopausal women. First, we tested the effects of the GRP30 agonist G1 on learning and memory in OVX mice (Supplemental Figure 1A; supplemental material available online with this article; https://doi.org/10.1172/JCI165812DS1). One week after ovariectomy, G1 was s.c. injected into the dorsal side of the neck once daily for 1 week, and then the novel object recognition (NOR) and fear conditioning (FC) tests were performed. Compared with the control mice, OVX mice exhibited a decrease in the discrimination index (Supplemental Figure 1, B–D) and freezing time (Supplemental Figure 1, E–H) in the NOR test and the contextual memory test, respectively, and these effects were reversed by G1 treatment in a dose-dependent manner, indicating that GPR30 agonists protect against memory impairment in OVX mice.

### Loss of GPR30 in astrocytes induced learning and memory deficits.

To test the cell-specific function of GPR30 in learning and memory, CRISPR/Cas9 technology was used to generate global GPR30–KO mice (Δ3102) (Supplemental Figure 2A). To obtain lineage-specific KO mice, we generated mice expressing a floxed GPR30 allele (GPR30*^fl/fl^*) and intercrossed them with mouse glial fibrillary acidic protein (GFAP, an astrocyte marker)- or neuron-specific enolase (NSE, a neural marker)-specific Cre transgenic (GFAP-Cre and NSE-Cre) mice to produce astrocyte-specific GPR30 KO (AG-KO, GPR30*^fl/fl^*;GFAP-Cre) and neuron-specific GPR30 KO (NG-KO, GPR30*^fl/fl^*;NSE-Cre) mice (Supplemental Figure 2B). The characteristics of global GPR30-KO and lineage-specific–KO mice have been reported previously (31). In this study, we performed immunostaining after fluorescence in situ hybridization (FISH) and confirmed the deletion of *GPR30* mRNA in hippocampal astrocytes and neurons of AG-KO and NG-KO mice, respectively (Supplemental Figure 2, C and D).

**Figure 1:**
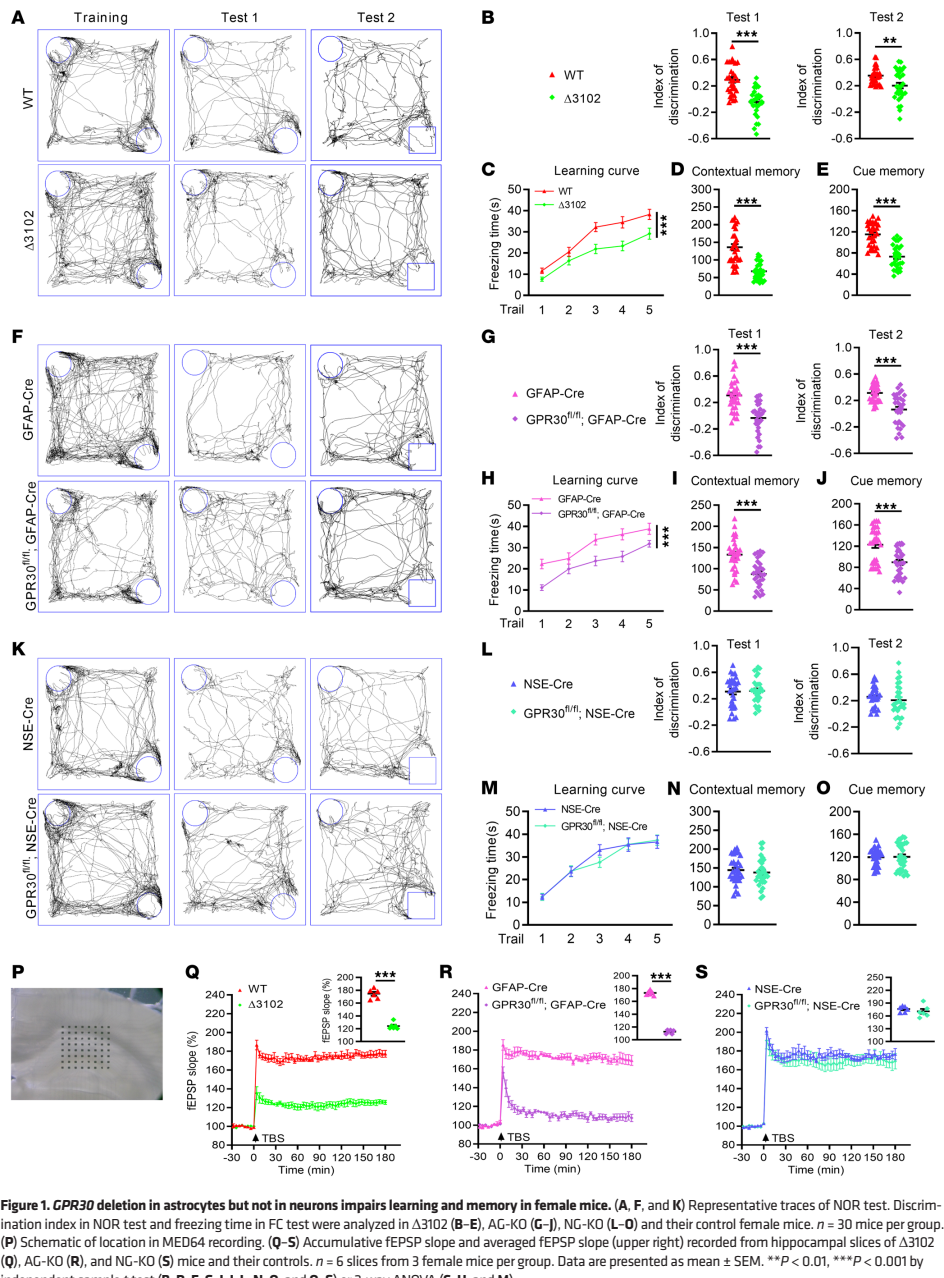
*GPR30* deletion in astrocytes but not in neurons impairs learning and memory in female mice. (**A**, **F**, and **K**) Representative traces of NOR test. Discrimination index in NOR test and freezing time in FC test were analyzed in Δ3102 (**B**–**E**), AG-KO (**G**–**J**), NG-KO (**L**–**O**) and their control female mice. *n* = 30 mice per group. (**P**) Schematic of location in MED64 recording. (**Q**–**S**) Accumulative fEPSP slope and averaged fEPSP slope (upper right) recorded from hippocampal slices of Δ3102 (**Q**), AG-KO (**R**), and NG-KO (**S**) mice and their controls. *n* = 6 slices from 3 female mice per group. Data are presented as mean ± SEM. **P < 0.01, ***P < 0.001 by independent sample *t* test (**B**, **D**, **E**, **G**, **I**, **J**, **L**, **N**, **O**, and **Q**–**S**) or 2-way ANOVA (**C**, **H**, and **M**).

Behavioral tests were performed during the 10th postnatal week. Δ3102 and AG-KO female mice showed a lower discrimination index in the NOR test (Figure 1, A, B, F, and G) and less freezing time in the FC test (Figure 1, C–E and H–J) than their controls. Interestingly, GPR30 deletion in neurons did not influence behavior (Figure 1, K–O). These results indicate that astrocytic GPR30 depletion caused learning and memory deficits in female mice. However, none of the male mice exhibited obvious changes in behavior (Supplemental Figure 3), suggesting that GPR30 plays a more important role in learning and memory in female mice than in male mice. Therefore, female mice were predominantly used in subsequent experiments.

To confirm the effect of astrocytic GPR30 on learning and memory, a 64-channel multielectrode (MED64) system was used to assess long-term potentiation (LTP) in the hippocampus, which is thought to underlie learning and memory (32). LTP was induced in the CA1 area by theta burst stimulation (TBS) of the Schaffer collateral pathway (Figure 1P). There was a dramatic decrease in LTP in Δ3102 and AG-KO female mice compared with controls (Figure 1, Q and R) but no change in LTP in NG-KO mice (Figure 1S). Furthermore, G1 (200 ng/μL, 100 μL/mouse) was s.c. injected to AG-KO female mice, but both the discrimination index and freezing time were not improved (Supplemental Figure 4). The data indicate that GPR30 in astrocytes, but not in neurons, is required to maintain normal learning and memory in female mice.

### Astrocytic GPR30 depletion led to neuronal and astrocytic abnormalities.

**Figure 2:**
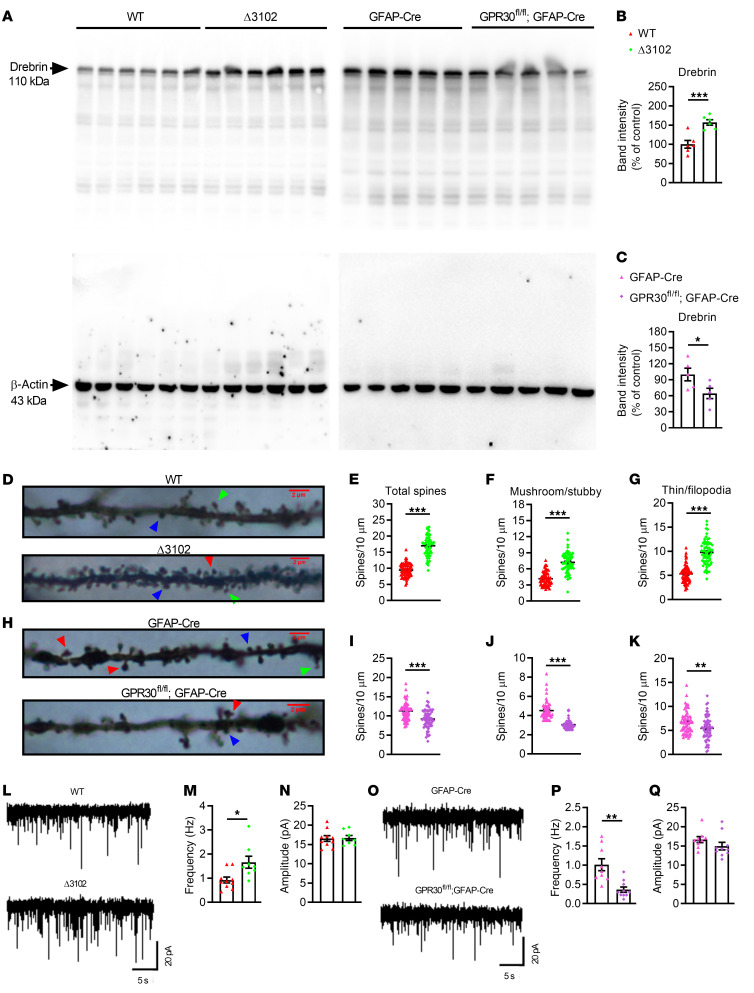
Deletion of *GPR30* in astrocytes induces neuronal abnormalities. (**A**–**C**) Immunoblots of Drebrin in hippocampal protein lysates (**A**). Quantification of Drebrin in Δ3102 (**B**, *n* = 6), AG-KO (**C**, *n* = 5), and their control female mice. (**D**–**K**) Basilar dendritic spines in Golgi-Cox-stained slices of the hippocampus (**D** and **H**). The blue arrowheads indicate stubby spines, the red arrowheads indicate mushroom spines, and the green arrowheads indicate thin/filopodia spines. Scale bar: 2 μm. Quantification of total, mushroom/stubby, and thin/filopodia spines in the hippocampi of Δ3102 (**E**–**G**) and AG-KO mice (**I**–**K**). *n* = 65 neurons from 5 female mice per group. (**L**–**Q**) Representative traces of sEPSCs in CA1 hippocampal pyramidal neurons from Δ3102 (**L**) and AG-KO female mice (**O**). Quantification of the sEPSCs frequency and amplitude in Δ3102 (**M** and **N**, *n* = 10 neurons from 3 WT mice; *n* = 8 neurons from 3 Δ3102 mice) and AG-KO mice (**P** and **Q**, *n* = 9 neurons from 3 control mice; *n* = 10 neurons from 3 AG-KO mice). Data are presented as mean ± SEM. *P < 0.05, **P < 0.01, ***P < 0.001 by independent sample *t* test.

Impairment of learning and memory is related to abnormalities in neuronal spines. Therefore, we detected the dendritic spine marker Drebrin in the hippocampi of 10-week-old female mice. The levels of Drebrin were significantly increased in Δ3102 mice (Figure 2, A and B) but decreased in AG-KO mice (Figure 2, A and C) compared with their controls. The levels of Drebrin were not changed in NG-KO mice (Supplemental Figure 5A). Then, we analyzed the dendritic spine density of CA1 hippocampal neurons by Golgi-Cox staining. In line with the above results, the total number of dendritic spines was increased in Δ3102 female mice (Figure 2, D and E) and decreased in AG-KO female mice (Figure 2, H and I). Spines are typically classified into 4 types according to morphology: filopodia, thin, stubby, and mushroom spines (33). Thin and filopodia spines are thinner protrusions with no clear head, harbor a small postsynaptic density, and are considered immature spines, while mushroom and stubby spines are thought to be stable (containing the highest number of AMPA receptors) and mature spines (34). Both mature and immature spine densities were elevated in Δ3102 mice (Figure 2, F and G) but decreased in AG-KO mice (Figure 2, J and K).Dendritic spines are the basic functional units of neuronal integration and are closely related to synaptic transmission (35).

Next, whole-cell patch-clamp recordings were performed in female CA1 pyramidal neurons from acute brain slices to record spontaneous excitatory postsynaptic currents (sEPSCs). The frequency of sEPSCs was significantly increased in Δ3102 mice (Figure 2, L and M) and reduced in AG-KO mice (Figure 2, O and P); however, the amplitude of sEPSCs was unchanged (Figure 2, N and Q). We also found that both the frequency and amplitude of sEPSCs were increased in NG-KO mice (Supplemental Figure 5, B and C). The differences in the pathological changes between Δ3102 and AG-KO mice may be attributed to microglial dysfunction in Δ3102 mice. As we reported previously, GPR30 can regulate microglial phagocytosis (36), which plays a key role in dendritic spine pruning (37).

**Figure 3:**
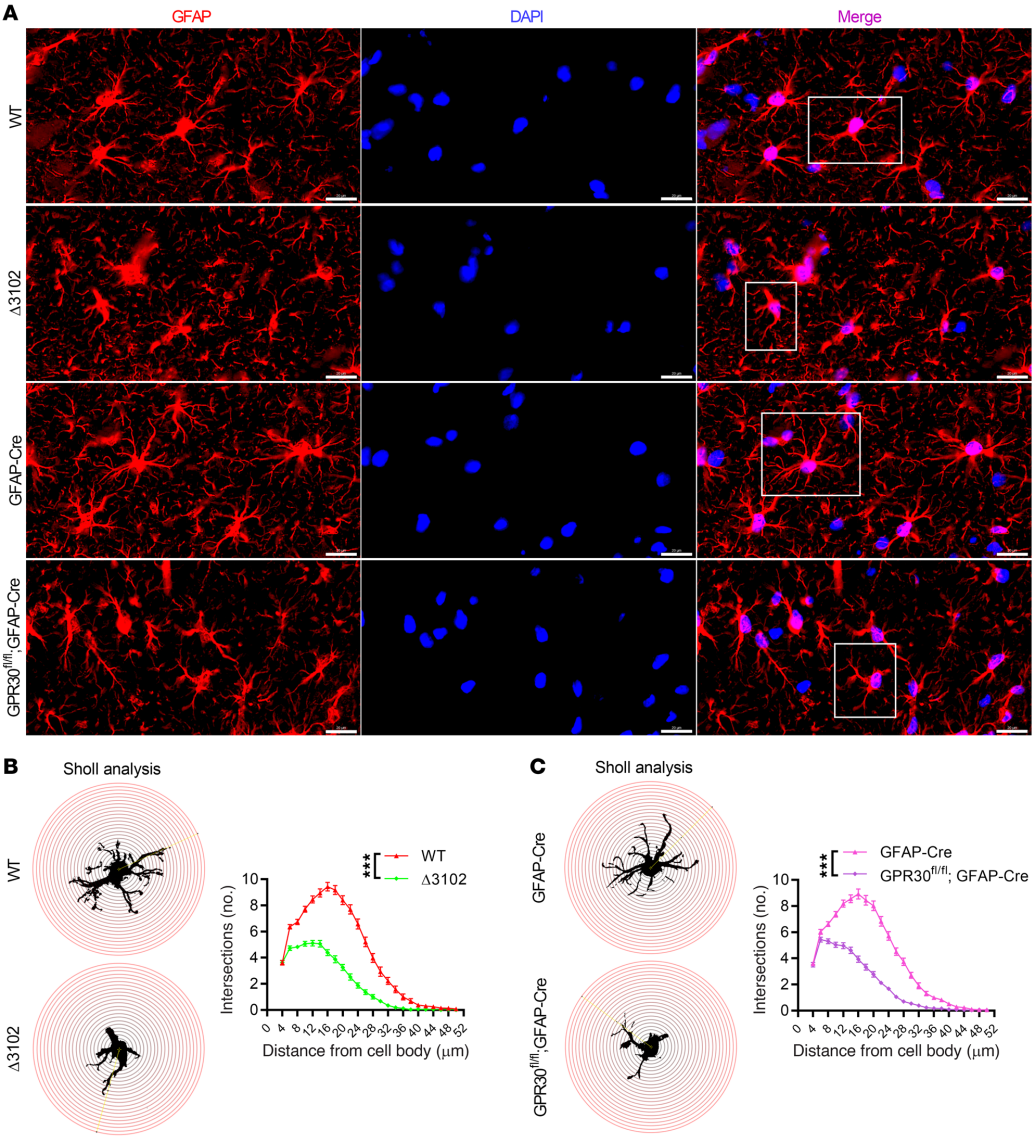
Deletion of *GPR30* in astrocytes simplifies astrocyte morphology. (**A**) Confocal images of GFAP-stained hippocampal slices from Δ3102 and AG-KO female mice. Scale bar: 20 μm. White box indicates astrocyte further examined in **B** and **C**. (**B** and **C**) Scholl analysis of astrocyte complexity in Δ3102 (B) and AG-KO mice (**C**). *n* = 100 cells from 5 mice per group. Data are presented as mean ± SEM. ***P < 0.001 by 2-way ANOVA.

Neuronal dysfunction is caused by astrocyte function impairment in AG-KO mice. Therefore, we evaluated changes in the complexity of astrocyte morphology, which is essential for astrocytic function in the brain (16, 38). Staining for astrocyte marker GFAP and high-resolution confocal imaging were performed. Sholl analysis of astrocytes in the female CA1 region revealed reduced morphological complexity in both Δ3102 and AG-KO mice (Figure 3). Astrocytes from NG-KO mice exhibited normal morphology (Supplemental Figure 5, F and G). The cell body area of astrocytes was not different among the groups (Supplemental Figure 5, D, E, and H). As the physiological activities of astrocytes depend on their morphological complexity, we further examined Ca^2+^ activity in astrocytes in the CA1 region during the training phase of the FC test. AAV5-GfaABC1D-GCaMP6f was injected into the CA1 region of the hippocampus, followed by the insertion of an optic fiber. The calcium indicator GCaMP6f was specifically expressed in astrocytes under the control of the GfaABC1D promoter (Supplemental Figure 6A). Ca^2+^ activity was recorded 4 weeks after virus injection. The Ca^2+^ signal intensity was much lower in AG-KO mice than in control mice after foot shock (Supplemental Figure 6, B and C and Supplemental Video 1). Together, these studies indicate that GPR30 deletion in astrocytes resulted in aberrant changes in astrocytic morphology and impairment of physiological functions and neuronal plasticity in the hippocampus of female mice.

### Acute astrocytic GPR30 KO in the CA1 area induced memory loss and imbalance of A1/A2 astrocytes.

**Figure 4:**
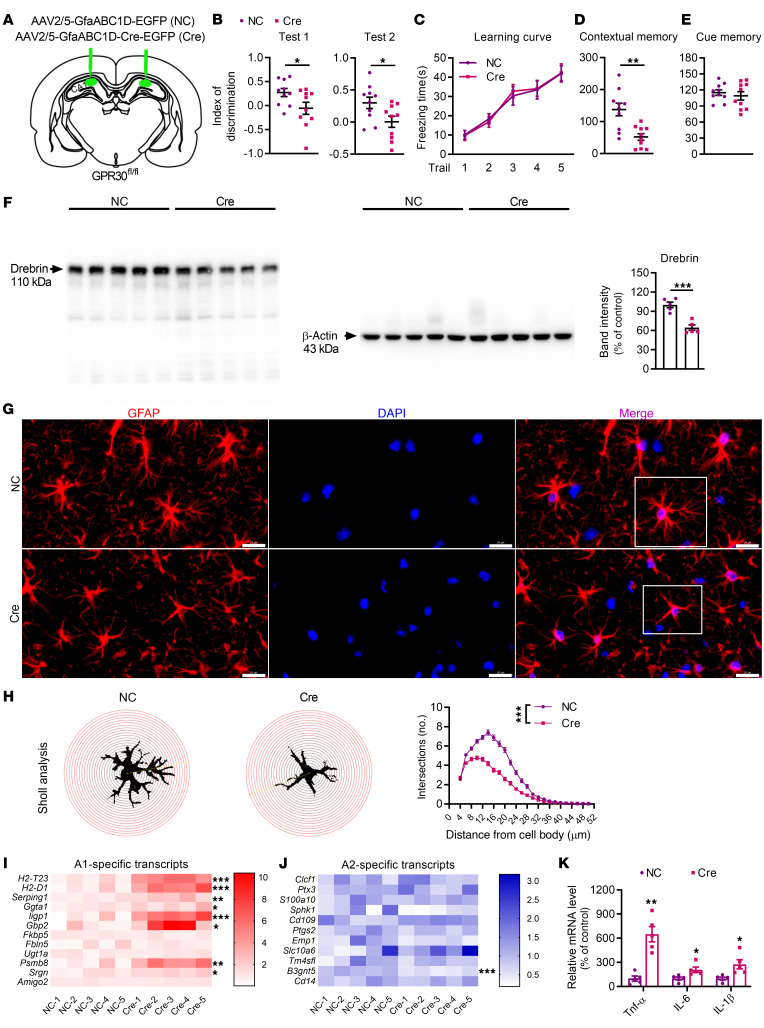
GPR30 KO in mature astrocytes in the CA1 results in impairment of memory and A1 polarization of astrocytes. (**A**) Diagram of virus injection into the CA1 region in the coronal plane. (**B**–**E**) Discrimination index in NOR test and freezing time in FC test were analyzed in NC and Cre mice. *n* = 10 mice per group. (**F**) Immunoblots of hippocampal protein lysates and quantification of Drebrin in NC and Cre mice. *n* = 5 mice per group. (**G** and **H**) Representative images of GFAP immunostaining (white box indicates astrocyte further examined in **H**) (**G**) and Sholl analysis of astrocyte complexity in the hippocampi of NC and Cre mice (**H**). *n* = 100 cells from 3 mice per group. Scale bar: 20 μm. (**I** and **J**) Heatmap of the levels of A1 (**I**) and A2 (**J**) astrocyte marker genes in NC and Cre mice. *n* = 5 mice per group. (**K**) Relative mRNA levels of *TNF-*α, *IL-6*, and *IL-1*β in the hippocampi of NC and Cre mice. *n* = 5 mice per group. NC indicates control mice. Cre indicates astrocytic GPR30–KO mice. Data are presented as mean ± SEM. *P < 0.05, **P < 0.01, ***P < 0.001 by independent sample *t* test (**B**, **D**–**F**, and **I**–**K**) or 2-way ANOVA (**C** and **H**).

To exclude the influence of GPR30 deletion on brain development in transgenic mice, AAV2/5-GfaABC1D-Cre-EGFP was bilaterally injected into the CA1 region in sixth postnatal–week GPR30*^fl/fl^* female mice to acutely delete astrocytic GPR30. In these mice (Cre mice), Cre recombinase was specifically expressed in astrocytes under the control of the GfaABC1D promoter, leading to bilateral astrocytic GPR30 depletion in the CA1 region (Figure 4A and Supplemental Figure 7A). Behavioral tests were performed during the tenth postnatal week. The discrimination index (Figure 4B) and contextual fear responses (Figure 4, C–E) were decreased in Cre mice with astrocytic GPR30 deletion compared to mice injected with negative control AAV (NC mice). The levels of Drebrin (Figure 4F) and morphological complexity of astrocytes (Figure 4, G and H) were also reduced in the hippocampi of Cre mice. Nevertheless, the cell body area of astrocytes remained unchanged (Supplemental Figure 7B).

To determine the changes in gene expression associated with GPR30 loss in astrocytes, quantitative real-time PCR (qRT–PCR) was performed to measure the levels of A1 and A2 astrocyte markers in the hippocampi of Cre and NC mice (19, 20). A1 astrocyte markers, including *H2-T23*, *H2-D1*, *Serping1*, *Ggta1*, *ligp1*, *Gbp 2*, *Psmb8*, and *Srgn*, were upregulated (Figure 4I), while A2 astrocyte markers, such as *Sphk1*, *Emp1*, and *B3gnt5*, were downregulated (Figure 4J) in Cre mice with astrocytic GPR30 deletion compared with NC mice. A1 astrocytes are closely associated with neuroinflammation and neurotoxicity (20). Consistently, the mRNA levels of the proinflammatory cytokines *TNF-*α, *IL-6,* and *IL-1*β were apparently higher in the hippocampi of Cre mice than in the hippocampi of NC mice (Figure 4K).

**Figure 5:**
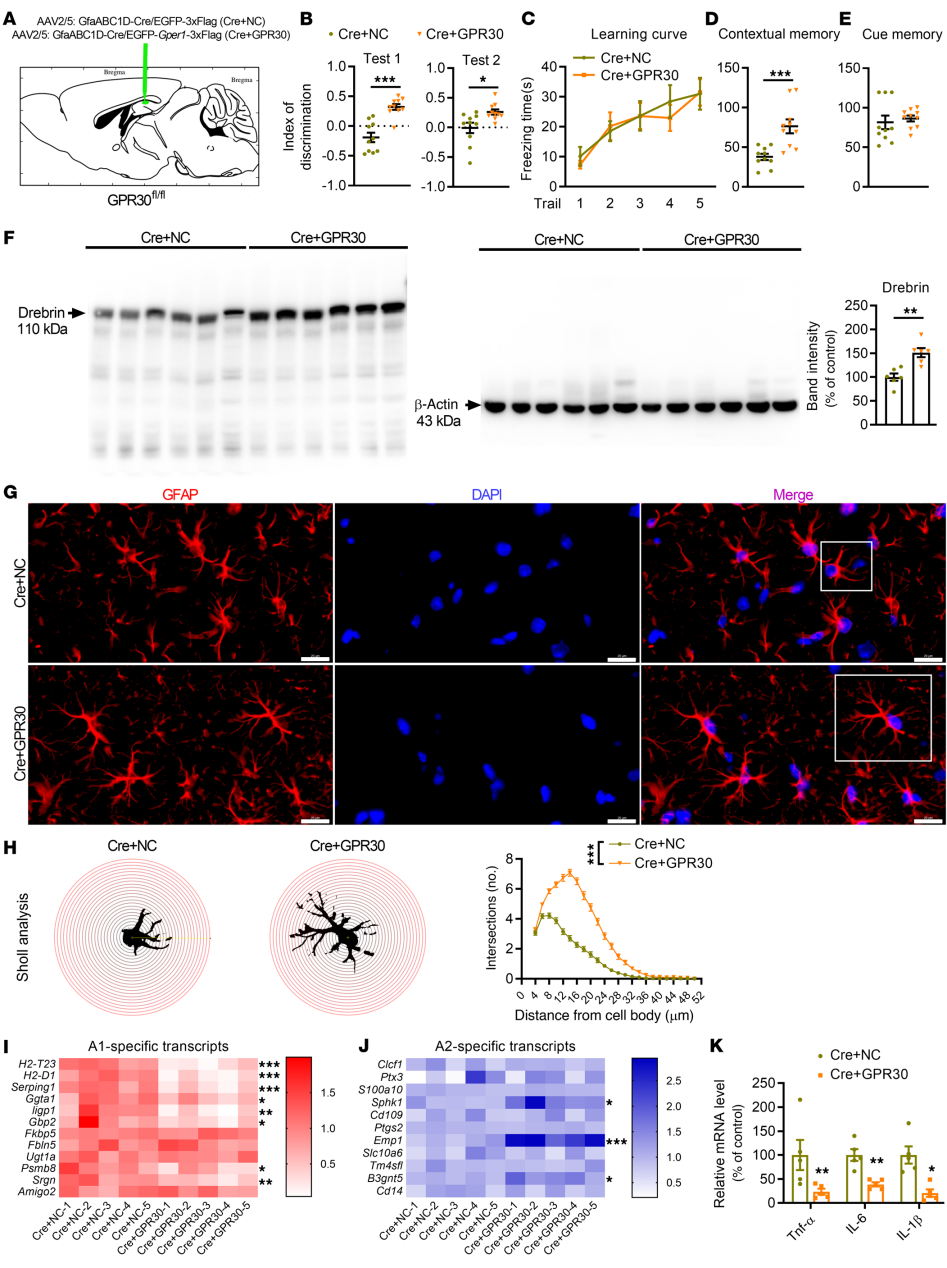
Restoration of GPR30 expression in astrocytes rescues astrocyte function impairment. (**A**) Diagram of the injection location in the sagittal plane. (**B**–**E**) Discrimination index in NOR test and freezing time in FC test were analyzed in Cre+NC and Cre+GPR30 mice. *n* = 10 mice per group. (**F**) Immunoblots of Drebrin in the hippocampi of Cre+NC and Cre+GPR30 mice. *n* = 6 mice per group. (**G** and **H**) Representative images of GFAP immunostaining (white box indicates astrocyte further examined in **H**) (**G**) and Sholl analysis of astrocyte complexity in the hippocampi of Cre+NC and Cre+GPR30 mice (**H**). *n* = 100 cells from 3 mice per group. Scale bar: 20 μm. (**I** and **J**) Heatmap of the expression levels of A1 (**I**) and A2 (**J**) astrocyte marker genes in Cre+NC and Cre+GPR30 mice. *n* = 5 mice per group. (**K**) Relative mRNA levels of *TNF-*α, *IL-6*, and *IL-1*β in the hippocampi of Cre+NC and Cre+GPR30 mice. *n* = 5 mice per group. Cre+NC indicates astrocytic GPR30–KO mice. Cre+GPR30 indicates mice with astrocytic GPR30 KO and restoration of GPR30 expression. Data are presented as mean ± SEM. *P < 0.05, **P < 0.01, ***P < 0.001 by independent sample t test (**B**, **D**–**F**, and **I**–**K**) or 2-way ANOVA (**C** and **H**).

To further evaluate the role of astrocytic GPR30 in A1 and A2 astrocytes, we restored GPR30 expression in astrocytes of Cre mice (Figure 5A and Supplemental Figure 7C). AAV2/5-GfaABC1D-EGFP-*Gper1*-3xFlag and AAV2/5-GfaABC1D-EGFP-3xFlag carried the EGFP sequence, while AAV2/5-GfaABC1D-Cre did not carry EGFP. AAV2/5-GfaABC1D-Cre/AAV2/5-GfaABC1D-EGFP-*Gper1*-3xFlag (Cre+GPR30) or AAV2/5-GfaABC1D-Cre/AAV2/5-GfaABC1D-EGFP-3xFlag (Cre+NC) was injected into the CA1 region in GPR30*^fl/fl^* mice. Restoration of GPR30 expression in astrocytes rescued behavioral deficits (Figure 5, B–E), the reduction in Drebrin expression (Figure 5F), and the changes in astrocyte morphology (Figure 5, G and H and Supplemental Figure 7D). Moreover, restoration of GPR30 expression reestablished the balance between A1 and A2 astrocytes (Figure 5, I and J) and decreased the mRNA levels of *TNF-*α, *IL-6,* and *IL-1*β (Figure 5K). These data suggest that astrocytic GPR30 regulates A1/A2 polarization, which is important for the physiological function of astrocytes.

### PJA1 mediated the modulatory effects of astrocytic GPR30 on learning and memory.

**Figure 6:**
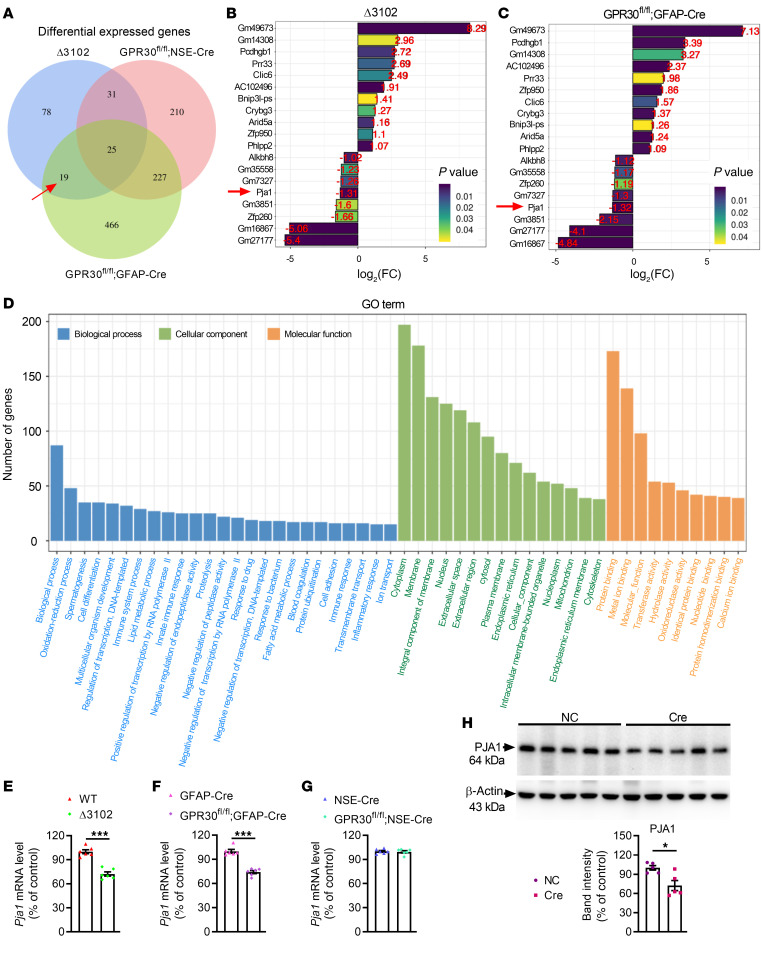
High-throughput RNA-Seq indicates a potential role of PJA1 in GPR30-deficient astrocytes. (**A**) Venn diagram depicting the overlapping and unique differentially expressed genes in the hippocampi of Δ3102, AG-KO, and NG-KO mice. (**B** and **C**) The fold changes (log_2_(FC)) and *P* values of the 19 identified differentially expressed genes are shown. (**D**) GO terms for which the differentially expressed genes in the hippocampi of AG-KO mice were enriched. (**E**–**G**) qPCR confirmed the expression levels of PJA1 in the hippocampi of Δ3102 (**E**), AG-KO (**F**), and NG-KO mice (**G**). *n* = 6 mice per group. (**H**) Cre-induced GPR30 deletion in astrocytes reduced PJA1 expression. *n* = 5 mice per group. NC indicates control mice. Cre indicates astrocytic GPR30–KO mice. Data are presented as mean ± SEM. *P < 0.05, ***P < 0.001 by independent sample *t* test.

To further elucidate the signaling pathway by which GPR30 plays a role in astrocyte function, high-throughput RNA-Seq of hippocampal tissues from Δ3102, AG-KO, and NG-KO female mice was performed. Overlapping differentially expressed genes in Δ3102 and AG-KO mice, excluding the differentially expressed genes in NG-KO mice, were selected. A total of 19 differentially expressed genes were identified (Figure 6A). The changing trend of the expression of these genes was similar in Δ3102 and AG-KO mice (Figure 6, B and C). Gene Ontology (GO) enrichment analysis revealed that the differentially expressed genes in AG-KO mice were mostly enriched in biological process-, cytoplasm-, and protein binding–related terms (Figure 6D). Accordingly, we focused PJA1 (Figure 6, B and C), an E3 ubiquitin ligase involved in neurodevelopmental disorders (28), which was significantly and stably downregulated. The expression of PJA1 in the hippocampi of Δ3102, AG-KO, and NG-KO mice was confirmed by qRT–PCR (Figure 6, E–G) and Western blotting (Supplemental Figure 8, A–D). PJA1 expression was reduced in Cre mice (Figure 6H) and increased upon restoration of GPR30 expression in astrocytes (Supplemental Figure 8E). However, the levels of PJA1 were unchanged in the hippocampi of AG-KO male mice (Supplemental Figure 8F).

**Figure 7:**
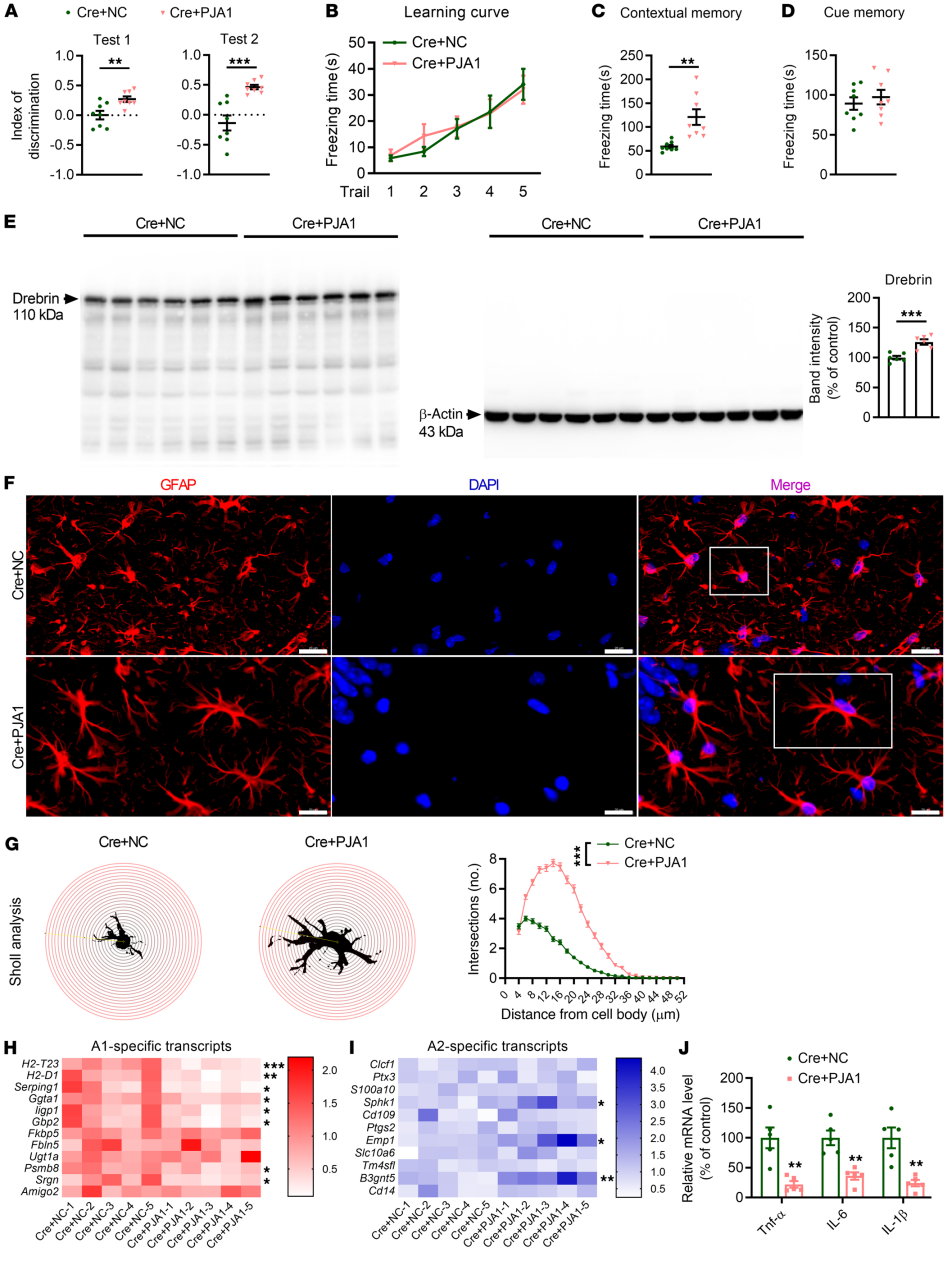
PJA1 mediates the effects of astrocytic GPR30 on learning and memory. (**A**–**D**) Discrimination index in NOR test and freezing time in FC test were analyzed in Cre+NC and Cre+PJA1 mice. *n* = 8 mice per group. (**E**) The Drebrin level was increased in the hippocampi of Cre+PJA1 mice. *n* = 6 mice per group. (**F** and **G**) Representative images of GFAP immunostaining (white box indicates astrocyte further examined in **G**) (**F**) and Sholl analysis of astrocyte complexity in the hippocampi of Cre+NC and Cre+PJA1 mice (**G**) *n* = 100 cells from 3 mice per group. Scale bar: 20 μm. (**H** and **I**) Heatmap of the levels of A1 (**H**) and A2 (**I**) astrocyte marker genes in Cre+NC and Cre+PJA1 mice. *n* = 5 mice per group. (**J**) Relative mRNA levels of *TNF-*α, *IL-6*, and *IL-1*β in the hippocampi of Cre+NC and Cre+PJA1 mice. *n* = 5 mice per group. Cre+NC indicates astrocytic GPR30–KO mice. Cre+PJA1 indicates astrocytic GPR30–KO and PJA1-upregulation mice. Data are presented as mean ± SEM. *P < 0.05, **P < 0.01, ***P < 0.001 by independent sample *t* test (**A**, **C**–**E**, and **H**–**J**) or 2-way ANOVA (**B** and **G**).

Next, upregulation of PJA1 along with Cre-mediated GPR30 deletion in astrocytes were induced by injecting AAV2/5-GfaABC1D-Cre/AAV2/5-GfaABC1D-EGFP-*Pja1*-3xFlag (Cre+PJA1) into the hippocampi of GPR30*^fl/fl^* female mice (Supplemental Figure 9, A–C). Upregulation of PJA1 alleviated learning and memory deficits induced by astrocytic GPR30 deletion (Figure 7, A–D), increased the levels of Drebrin and complexity of astrocyte morphology in the hippocampus (Figure 7, E–G and Supplemental Figure 9D), promoted the transformation of A1 astrocytes to A2 astrocytes, and decreased the levels of proinflammatory cytokines (Figure 7, H–J). These results suggest a key role for PJA1 in the function of astrocytic GPR30 in female mice.

### GPR30 positively regulated PJA1 expression through the CREB signaling pathway in astrocytes.

**Figure 8:**
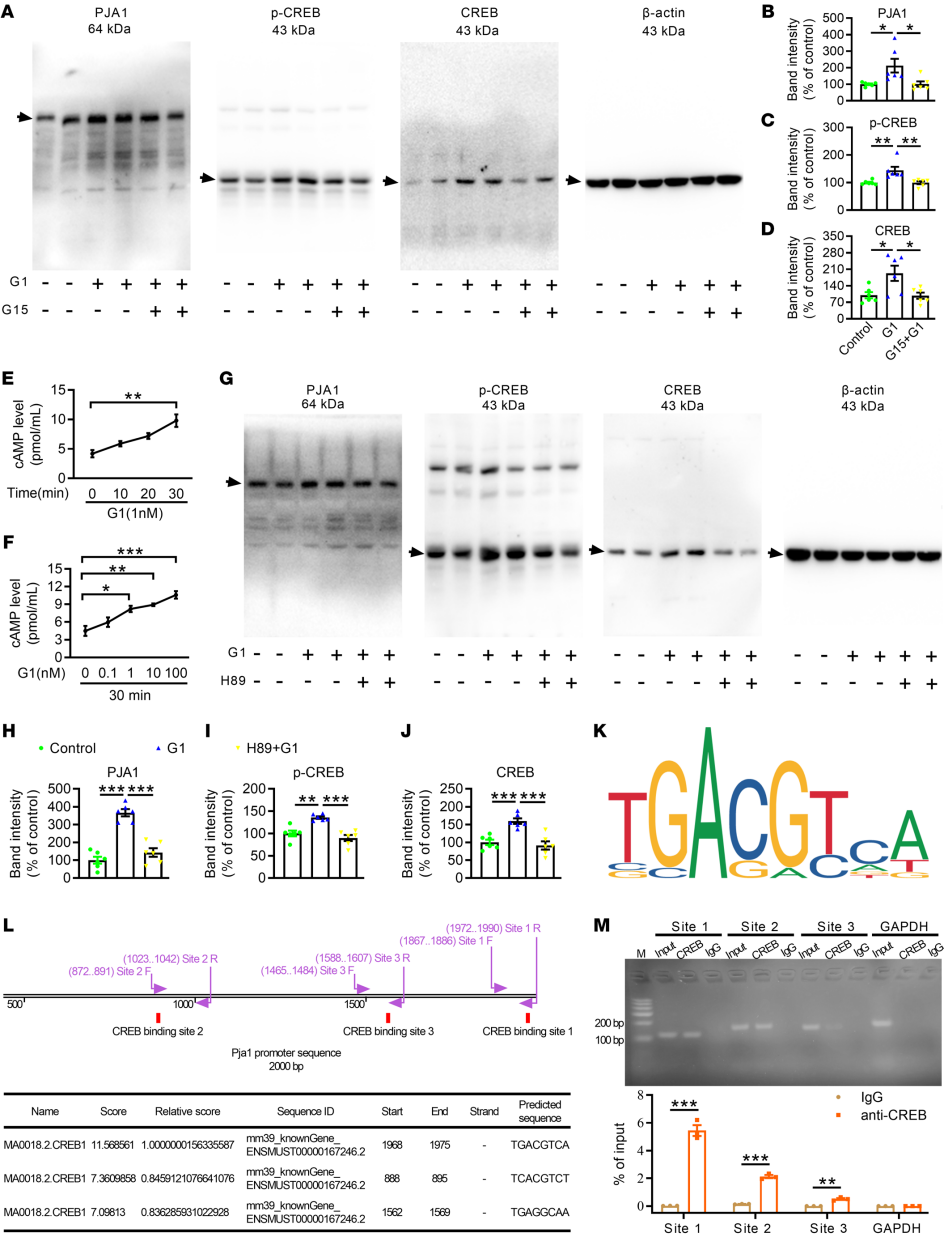
GPR30 regulates PJA1 expression through the CREB signaling pathway in astrocytes. (**A–D**) Representative immunoblots and quantification of PJA1 (**B**), p-CREB (**C**), and CREB (**D**) in cultured primary astrocytes treated with G1 and G15. *n* = 6 samples per group. (**E** and **F**) GPR30 activation increased cAMP levels in astrocytes in a time- and concentration-dependent manner. *n* = 3 samples per group. (**G**–**J**) Representative immunoblots and quantification of PJA1 (**H**), p-CREB (**I**), and CREB (**J**) in cultured astrocytes treated with G1 and H89. *n* = 6 samples per group. (**K**) Motif plot for CREB (http://jaspar.genereg.net/). (**L**) Bottom: The top 3 binding sites of CREB according to their predicted scores. Top: Primers designed to verify the binding of CREB. (**M**) The binding of CREB and IgG to the promoter region of *Pja1* in astrocytes, as determined by ChIP. The experiment was repeated 3 times. Data are presented as mean ± SEM. *P < 0.05, **P < 0.01, ***P < 0.001 by 1-way ANOVA with Tukey’s posthoc test (**B**–**F**, and **H**–**J**) or independent sample *t* test (**M**).

Next, primary astrocytes were cultured to further investigate the mechanism by which GPR30 regulates PJA1. Treatment with 1 nM of G1, a GPR30 agonist, significantly increased the protein levels of PJA1 in astrocytes, and this effect was inhibited by the GPR30 antagonist G15 (100 nM, Figure 8, A and B). It has been reported that GPR30 mediates nongenomic estrogenic responses by coupling to Gs (39) and Gi/o proteins (40). Thus, we analyzed the type of G protein coupled to GPR30 and found that cAMP levels increased in a time- and concentration-dependent manner after G1 treatment, suggesting that GPR30 may bind to Gs protein in astrocytes (Figure 8, E and F). Next, the levels of phosphorylated CREB (p-CREB) and CREB were measured after G1 treatment, as cAMP/PKA/CREB is a classic signaling pathway downstream of Gs protein (41). Both p-CREB levels and CREB levels were increased after G1 treatment, and this effect was blocked by G15 pretreatment (Figure 8, A, C, and D). The primary cells above were a mixture of female and male astrocytes. In order to clarify whether astrocytes from female and male mice respond differently to G1, the sex of newborn mice was determined by detecting the sex-determining region of Y-chromosome (Sry) gene (Supplemental Figure 10A), and primary astrocytes from different sexes were cultured separately. G1 treatment enhanced the expression of PJA1 and activated CREB signaling only in astrocytes of female mice but not in astrocytes of male mice (Supplemental Figure 10, B–I), indicating that the results of mixed astrocytes were similar to those of female astrocytes and that GPR30 selectively regulates PJA1 expression in female astrocytes.

To confirm the role of PKA/CREB signaling in the GPR30-mediated regulation of PJA1, the PKA inhibitor H89 was administered along with G1 to cultured astrocytes. H89 abolished the enhancement of PJA1, p-CREB, and CREB expression induced by G1 (Figure 8, G–J). Then, the JASPER database (http://jaspar.genereg.net/) was used to predict whether CREB can directly bind to the promoter of *Pja1*. The *Pja1* promoter sequence was obtained from the UCSC database (http://genome.ucsc.edu/) (Supplemental Figure 11). A CREB motif (Figure 8K, image from JASPAR database) was employed to predict CREB binding sites in the *Pja1* promoter. A higher predicted score indicates a higher binding probability. The top 3 binding sites were verified by ChIP experiments (Figure 8L). The data showed that CREB could bind to all 3 regions of the *Pja1* promoter but not to GAPDH (a negative control) in astrocytes (Figure 8M). These data suggest that GPR30 modulates PJA1 by activating the cAMP/PKA/CREB signaling pathway in astrocytes.

### Serpina3n was degraded by PJA1 in astrocytes and modulated learning and memory.

**Figure 9:**
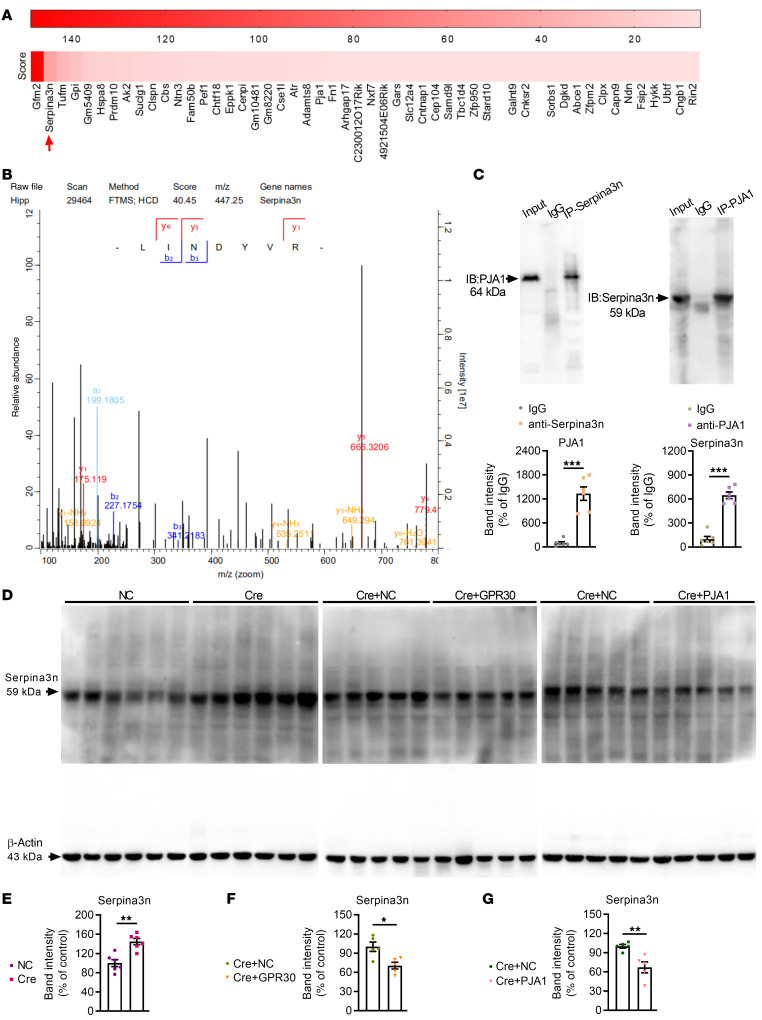
PJA1 modulates the degradation of Serpina3n. (**A**) The ranking of identified proteins specifically associated with PJA1 in the hippocampus based on scores calculated from the posterior error probabilities of the identified peptides. (**B**) Serpina3n-specific MS/MS spectra for peptide identification. (**C**) Co-IP showing the interaction between PJA1 and Serpina3n. (**D**–**G**) Immunoblots (**D**) and quantification of Serpina3n in the hippocampi of Cre (**E**, *n* = 6 mice per group), Cre+GPR30 (**F**, *n* = 5 mice per group) and Cre+PJA1 mice (**G**, *n* = 5 mice per group). NC indicates control mice. Cre/Cre+NC indicate astrocytic GPR30–KO mice. Cre+GPR30/PJA1 indicates astrocytic GPR30–KO and GPR30/PJA1-upregulation mice. Data are presented as mean ± SEM. *P < 0.05, **P < 0.01, ***P < 0.001 by independent sample *t* test.

PJA1, a RING E3, mediates the direct transfer of ubiquitin to the substrate. We speculated that PJA1 may influence the function of astrocytes by modulating the degradation of its binding substrates. Immunoprecipitation (IP) and mass spectrometry (MS) were employed to identify PJA1-interacting proteins in the hippocampus. The identified proteins were ranked according to the score calculated from the posterior error probabilities of the identified peptides (Figure 9A). A higher score indicated a higher probability that the identified protein interacts with PJA1. The first-ranked protein, GFM2 (ribosome-releasing factor 2, mitochondrial), is a mitochondrial translation elongation factor (42, 43). Its role in the regulation of normal mitochondrial function and mitochondrial dysfunction–induced diseases is still largely unknown. The second-ranked protein, Serpina3n (Figure 9B), is closely associated with neuroinflammation and cognition (44, 45). Serpina3n expression is especially elevated in astrocytes in the presence of neuroinflammation and in the aged brain (19, 46). Therefore, the interaction between PJA1 and Serpina3n was further confirmed by coimmunoprecipitation (Co-IP) (Figure 9C), and the results indicated that Serpina3n is a potential substrate of PJA1.

Then, we assessed the levels of Serpina3n in the hippocampus in each GPR30 transgenic mouse strain. We found that Serpina3n expression was increased in Δ3102 and AG-KO mice but unchanged in NG-KO female mice (Supplemental Figure 12, A–D). It was also significantly increased in the hippocampi of GPR30*^fl/fl^* female mice injected with Cre-expressing AAV (Figure 9, D and E). However, the levels of Serpina3n were decreased after restoration of GPR30 expression or induction of PJA1 upregulation (Figure 9, D, F and G). The Serpina3n levels were also unchanged in AG-KO male mice (Supplemental Figure 12E). These data suggest that GPR30 and PJA1 negatively modulate Serpina3n in female mice.

**Figure 10:**
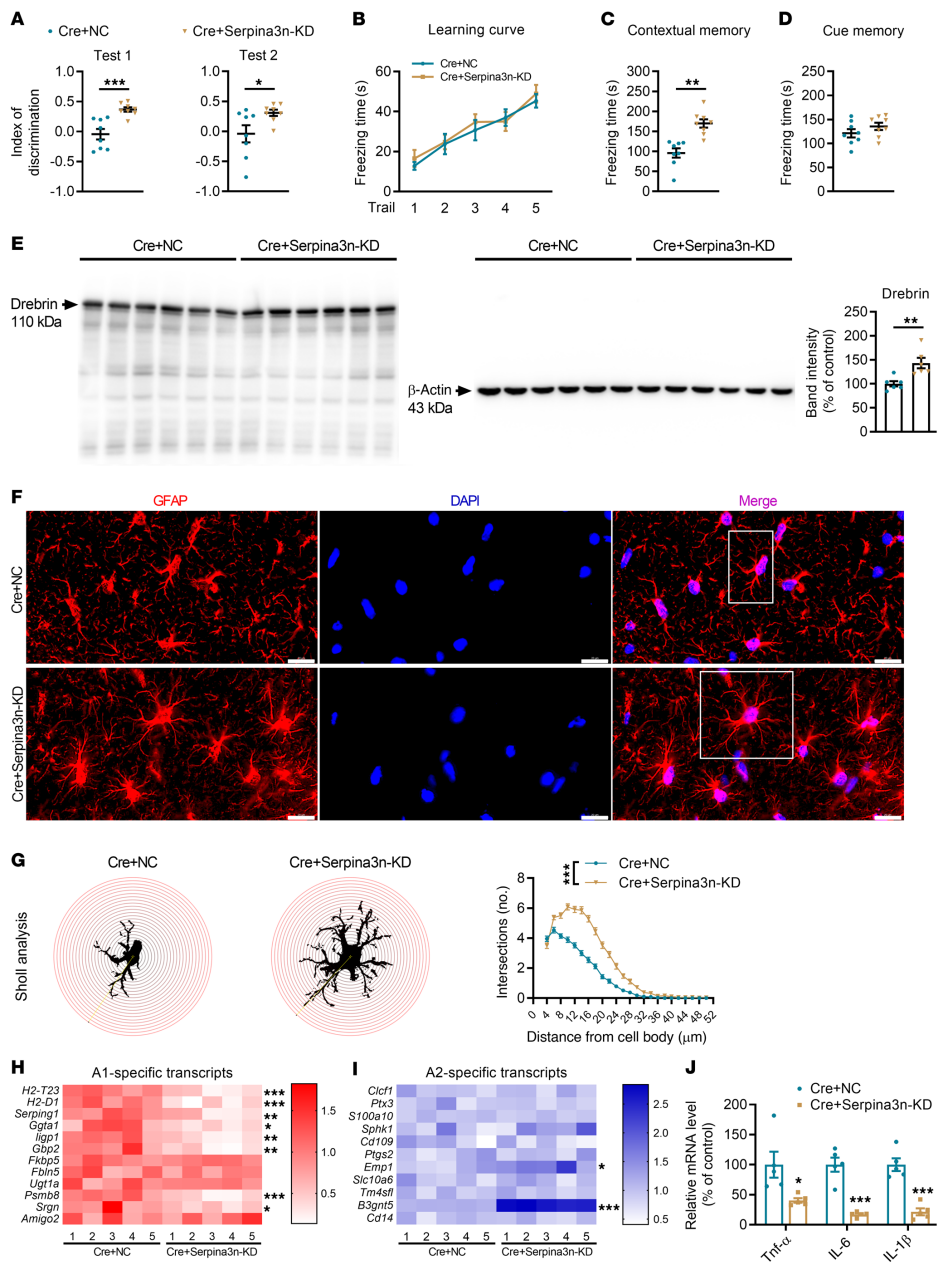
Serpina3n KD restores learning and memory of astrocytic GPR30–deletion mice. (**A**–**D**) Discrimination index in NOR test and freezing time in FC test were analyzed in Cre+NC and Cre+Serpina3n-KD mice. *n* = 8 mice per group. (**E**) The level of Drebrin was increased in the hippocampi of Cre+ Serpina3n-KD mice. *n* = 6 mice per group. (**F** and **G**) Images of GFAP immunostaining (white box indicates astrocyte further examined in **G**) (**F**) and Sholl analysis of astrocyte complexity in the hippocampi of Cre+NC and Cre+Serpina3n-KD mice (**G**). *n* = 100 cells from 3 mice per group. Scale bar: 20 μm. (**H** and **I**) Heatmap of the levels of the A1 (**H**) and A2 (**I**) astrocyte marker genes in Cre+NC and Cre+Serpina3n-KD mice. *n* = 5 mice per group. (**J**) Relative mRNA levels of *TNF-*α, *IL-6*, and *IL-1*β in the hippocampi of Cre+NC and Cre+Serpina3n-KD mice. *n* = 5 mice per group. Cre+NC indicates astrocytic GPR30–KO mice. Cre+ Serpina3n-KD indicates astrocytic GPR30 KO and Serpina3n-KD mice. Data are presented as mean ± SEM. *P < 0.05, **P < 0.01, ***P < 0.001 by independent sample *t* test (**A**, **C**–**E**, and **H**–**J**) or 2-way ANOVA (**B** and **G**).

To further test the role of Serpina3n in astrocytes, Serpina3n was knocked down and GPR30 was deleted in astrocytes in the female CA1 region (Cre+Serpina3n–KD mice, Supplemental Figure 13, A–C). Serpina3n knockdown (KD) restored learning and memory abilities (Figure 10, A–D) and rescued the decreases in the levels of Drebrin and complexity of astrocyte morphology in the hippocampus induced by GPR30 deletion in astrocytes (Figure 10, E–G and Supplemental Figure 13D). KD of Serpina3n also promoted the transformation of A1 astrocytes to A2 astrocytes and reduced the levels of *TNF-*α, *IL-6*, and *IL-1*β (Figure 10, H–J). These data suggest that impairment of PJA1-mediated Serpina3n degradation resulted in astrocyte activation and proinflammatory cytokine release, which impair synaptic plasticity, learning, and memory in female mice.

### GPR30/PJA1/Serpina3n signaling played key roles in the learning and memory abilities of OVX mice and menopausal women.

**Figure 11:**
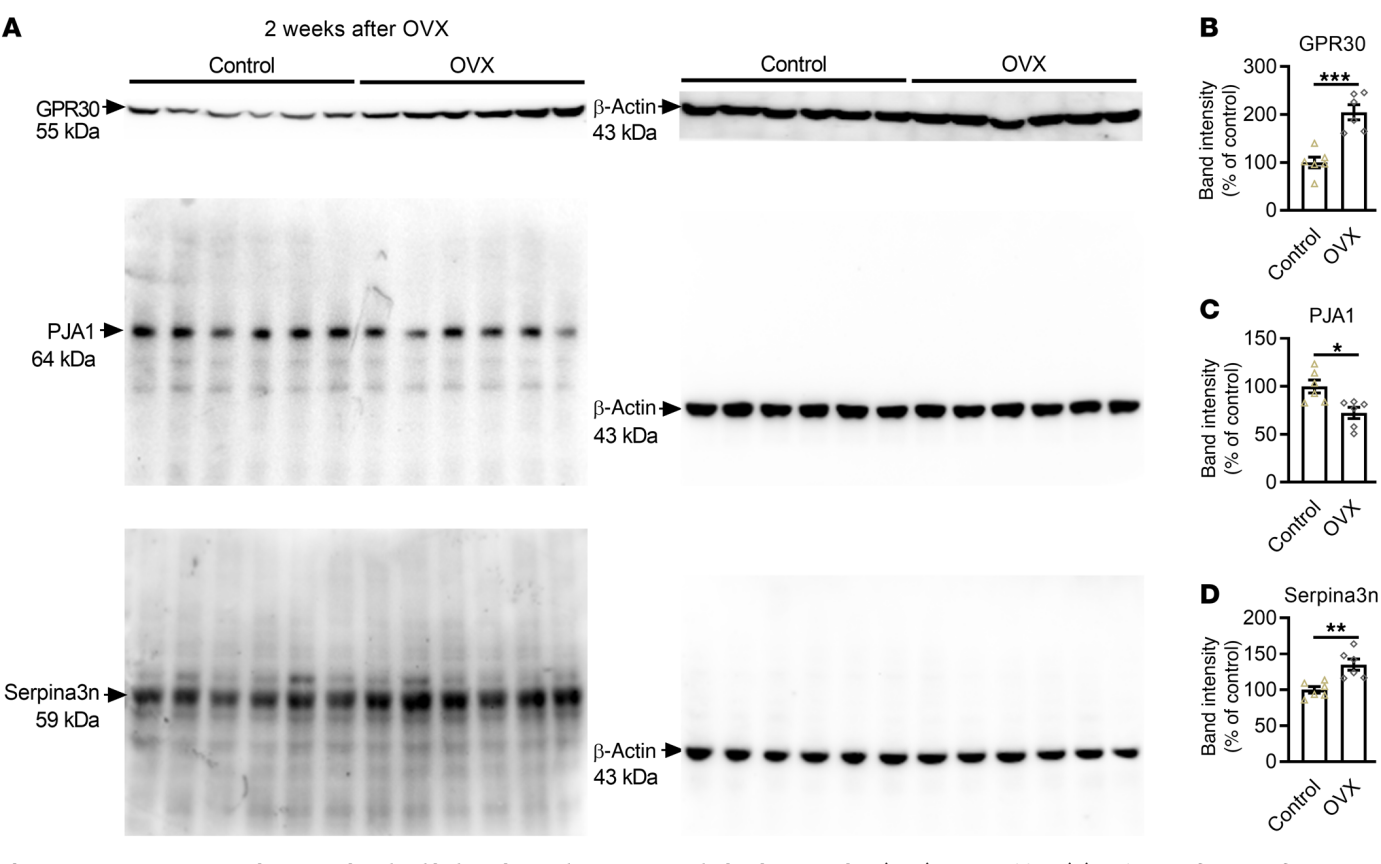
GPR30/PJA1/Serpina3n are involved in learning and memory regulation in OVX mice. (**A**–**D**) Immunoblots (**A**) and quantification of GPR30 (**B**), PJA1 (**C**), and Serpina3n (**D**) in the hippocampus. *n* = 6 mice per group. Data are presented as mean ± SEM. *P < 0.05, **P < 0.01, ***P < 0.001 by independent sample *t* test.

First, we verified the occurrence of this signaling alteration in the hippocampi of OVX mice. The level of GPR30 in the hippocampus was elevated 2 weeks after ovariectomy (Figure 11, A and B) but decreased 12 weeks after ovariectomy (Supplemental Figure 14A). Due to loss of estrogenic effects, PJA1 expression was significantly decreased both 2 weeks and 12 weeks after ovariectomy (Figure 11, A and C and Supplemental Figure 14, B and C), while Serpina3n expression was increased (Figure 11, A and D and Supplemental Figure 14, B and D). These data indicate the importance of GPR30/PJA1/Serpina3n signaling in the hippocampi of OVX mice.

**Figure 12:**
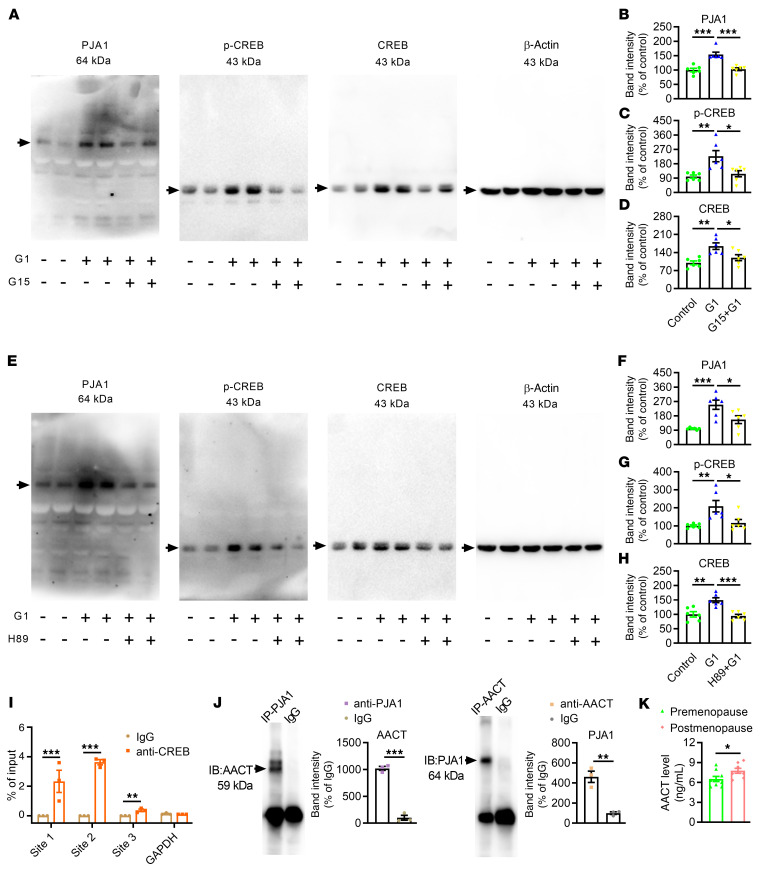
GPR30/PJA1/Serpina3n signaling pathway exists in human astrocytes. (**A**–**D**) Representative immunoblots (**A**) and quantification of PJA1 (**B**), p-CREB (**C**), and CREB (**D**) in normal human astrocytes (NHAs). *n* = 6 samples per group. (**E**–**H**) Representative immunoblots (**E**) and quantification of PJA1 (**F**), p-CREB (**G**), and CREB (**H**) after H89 treatment. *n* = 6 samples per group. (**I**) The binding of CREB to the promoter region of *Pja1* in NHAs was assessed by ChIP. The experiment was repeated 3 times. (**J**) Co-IP showing the interaction between PJA1 and AACT (a homolog of Serpina3n in humans). (**K**) The level of AACT in the plasma was measured by ELISA. *n* = 9 samples in the premenopausal group. *n* = 8 samples in the postmenopausal group. Data are presented as mean ± SEM. *P < 0.05, **P < 0.01, ***P < 0.001 by 1-way ANOVA with Tukey’s posthoc test (**B**–**D** and **F**–**H**) or independent sample *t* test (**I**–**K**).

Next, we confirmed the existence of this signaling pathway in cultured normal human astrocytes (NHAs) (Supplemental Figure 15A), which derive from female donor. Activation of GPR30 by G1 evidently enhanced the expression of PJA1 and activated CREB signaling (Figure 12, A–D), and these effects were inhibited by G15. H89 treatment abolished the regulatory effects of GPR30 on PJA1 and CREB signaling (Figure 12, E–H). Furthermore, ChIP revealed that CREB could directly bind to the *Pja1* promoter (Figure 12I, Supplemental Figure 15, B and C, and Supplemental Figure 16) and that PJA1 could directly interact with α1-antichymotrypsin (AACT, the human ortholog of murine Serpina3n) in NHAs (Figure 12J).

Finally, the mRNA levels of GPR30 and PJA1 were measured in exosomes isolated from the blood of premenopausal and postmenopausal women. Estrogen levels were significantly decreased in the plasma of postmenopausal women (Supplemental Figure 15D). Exosomes were isolated by ultracentrifugation and characterized by transmission electron microscopy (Supplemental Figure 15E). The levels of both *GPR30* and *PJA1* were reduced in postmenopausal women (Supplemental Figure 15F). However, the level of Serpina3n in the plasma was notably increased (Figure 12K). These results from mouse, human astrocyte, and blood samples reveal that the astrocytic GPR30/PJA1/Serpina3n signaling pathway plays a major role in learning and memory deficits during menopause.

## Discussion

The side effects of HRT limit its application for maintaining cognitive function or preventing cognitive decline in postmenopausal women (47, 48). In the present study, astrocytic *GPR30*–KO female mice had deficits in learning and memory. GPR30 deletion in astrocytes caused a molecular signature associated with A1-like reactive astrocytes, resulting in the release of the proinflammatory cytokines TNF-α, IL-6, and IL-1β, which damaged neurons. High-throughput RNA-Seq uncovered an important role for the RING E3 PJA1 — which binds with Serpina3n — in mediating the harmful outcomes of astrocytic GPR30 deficiency in female mice. Furthermore, we revealed that the regulatory effect of GPR30 on PJA1 depends on the cAMP/PKA/CREB signaling pathway. Finally, the existence of this signaling pathway was confirmed in NHAs and blood samples from postmenopausal women.

Several studies have shown that GPR30 activation or inhibition is involved in hippocampal-dependent memory such as spatial memory, social recognition, object recognition, and object placement recognition in OVX rats and mice (49–51). However, no studies have distinguished which type of cells mediate the effects of GPR30 on learning and memory. Our previous studies reported the neuroprotective effects of GPR30 in neurons, astrocytes, and microglia against excitotoxicity and oxidative injury (31, 36, 52, 53). Based on these studies, we found that GPR30 deletion in astrocytes — but not in neurons — causes learning and memory impairment in female mice. Male transgenic mice exhibited normal learning and memory, suggesting a key role for GPR30 in cognitive functions specifically in female mice, which is consistent with the higher level of GPR30 in the hippocampus in female mice compared with male mice (Supplemental Figure 17A). Behavioral function, LTP induction, and astrocyte morphology were similar between global GPR30–KO (Δ3102) and AG-KO female mice. However, the Drebrin level and density of neuronal spines were different between global GPR30–KO (Δ3102) and AG-KO mice. This may be attributed to the lack of neuronal pruning resulting from GPR30 loss in the microglia of Δ3102 mice. In contrast, astrocytic GPR30 deletion impairs the physiological function of astrocytes, which induces neuronal spine damage. This hypothesis needs to be explored in future studies. Here, we determined the role of astrocytic GPR30 in learning and memory of females.

Similar to activated microglia, which can be divided into M1 and M2 microglia, reactive astrocytes can be divided into A1 and A2 astrocytes. Lipopolysaccharide (LPS) promotes the polarization of astrocytes toward the deleterious A1 phenotype, and A1 astrocytes release more complement components and proinflammatory cytokines (20); hypoxia induces the polarization of astrocytes toward the A2 phenotype, and A2 astrocytes produce neurotrophic factors and antiinflammatory cytokines (19). Astrocytes with GPR30 deletion exhibited increases in the transcript levels of A1-specific genes as well as decreases in the transcript levels of A2-specific genes, and these changes were reversed by restoration of GPR30 expression, PJA1 upregulation, and Serpina3n knockdown. These data indicate that astrocytic GPR30 controls astrocytic conversion between the A1 and A2 phenotypes. Moreover, Sholl analysis of astrocytes from germline transgenic mice and mice injected with AAVs revealed similar results, suggesting a key role for GPR30 in the physiological function of astrocytes. Notably, not all A1- and A2-specific transcripts showed changes in levels in astrocytes with GPR30 deletion, as the levels of several transcripts remained constant under different treatment conditions. As reported, A1 astrocyte polarization has been proposed to be induced by activated microglia through microglia-derived TNF, IL-1α, and C1q (20). The A1 polarization of astrocytes with GPR30 deletion may not result from microglial activation but from the loss of astrocytic GPR30. The increases in the levels of proinflammatory cytokines (*TNF-*α, *IL-6*, and *IL-1*β) indicate that A1 astrocytes with GPR30 deletion are neurotoxic and impair neuronal plasticity.

We explored the mechanism underlying learning and memory deficits in astrocytic GPR30–KO mice, and high-throughput RNA-Seq revealed that PJA1, which is abundantly expressed in the human and mouse brain (27, 54), was deficient in these female mice. *Pja1*, a gene on the X chromosome, has been proposed as a candidate gene for mouse sex-linked sideroblastic anemia (54). Moreover, female mice showed higher expression of PJA1 in the hippocampus compared with male mice (Supplemental Figure 17B). This may be another reason for the lack of change in learning and memory abilities in male mice. The PJA1 protein belongs to a class of E3s that play central roles in ubiquitination. We thereby screened the potential binding targets of PJA1 by a combination of Co-IP and MS. We noted that some reported substrates of PJA1 in the brain did not appear in the analyzed list (Figure 6A) (28, 55, 56). In addition to the limitations of the experimental methods and systems, this could be attributed to differences in species, brain areas, and cell types. Finally, we focused on Serpina3n, a protein related to neuroinflammation (21, 44). Serpina3n expression has been reported to be upregulated in persistently active astrocytes and many neurological diseases (20, 44, 57), indicating that Serpina3n has a proinflammatory role. However, there are also conflicting results regarding Serpina3n. It has been reported that Serpina3n exerts neuroprotective effects (45, 58). Neuronal *Serpina3n* deficiency even aggravates learning and memory impairments in mice with hippocampal stab injury (45). Serpina3n may have different functions in neurons and astrocytes. As previously discussed, Serpina3n protects neurons against apoptosis after hippocampal injury but does not directly mediate inflammatory response of astrocytes (45). We speculate that Serpina3n deficiency may inhibit reactive astrogliosis and glial scar formation in the early stage of hippocampal stab injury. Overall, it is clear that Serpina3n accumulation plays a proinflammatory role in astrocytes.

Our current findings indicate that PJA1 mediates the ubiquitination-mediated degradation of Serpina3n in astrocytes. Loss of astrocytic GPR30 leads to a reduction in PJA1 expression and Serpina3n accumulation. Reversing either of these changes could rescue A1/A2 astrocyte polarization and learning and memory deficits in astrocytic GPR30-deficient mice. In addition, GPR30 and PJA1 mRNA levels were found to be much lower in blood exosomes from postmenopausal women than in those from premenopausal women. The level of Serpina3n in the blood was also increased in postmenopausal women. Although the changes in the blood cannot fully reflect those in the brain, these data at least indicate a potential association of GPR30/PJA1/Serpina3n with cognitive decline in postmenopausal women. Further, we confirmed the interaction between PJA1 and Serpina3n in NHAs derived from women. Together, the present study suggests that GPR30/PJA1/Serpina3n are potential drug targets for treating learning and memory loss during menopause.

GPCRs induce signal transduction by producing a number of second messengers, including cAMP. We found that GPR30 binds to Gs protein in cultured primary astrocytes. The Gs-cAMP/PKA/CREB pathway is a classical signaling pathway. GPR30 activation activated CREB signaling and promoted PJA1 expression, which was blocked by H89, a PKA inhibitor. These data verify that GPR30 regulates PJA1 expression via the cAMP/PKA/CREB signaling pathway. CREB regulates gene expression by binding to the cAMP response element (CRE) sequence, which is a conserved 8-bp palindromic sequence, i.e., TGACGTCA (59). By using computer tools, we screened several potential binding sites in the promoter of the *Pja1* gene. The 3 binding sites with the highest scores were validated by ChIP assay, indicating that CREB directly regulates *Pja1*. Here, we clarify the signaling pathway involved in the effect of astrocytic GPR30 on PJA1. Moreover, the existence of this regulatory pathway was confirmed in NHAs. However, we cannot completely rule out the contribution of other signaling pathways to the regulatory effect of astrocytic GPR30 on PJA1 expression and cognitive function.

In summary, our study emphasizes a major role for astrocytic GPR30 in learning and memory of female mice. The human and animal data point to GPR30/PJA1/Serpina3n as potential targets for treating learning and memory loss in perimenopause and postmenopause.

## Methods

### Animals.

The mice were housed under a 12-hour light/dark cycle with water and food provided ad libitum. The animal feeding room had a controlled temperature (22–26°C) and humidity (55%–60%). All mice were maintained on the C57BL/6J background. Normal female C57BL/6J mice aged 8 weeks were provided by the Laboratory Animal Center of the Fourth Military Medical University. Δ3102 *GPR30*–KO mice and GPR30*^fl/fl^* mice were obtained from Beijing Biocytogen Co, Ltd. Homozygous mice without any mutations were used as the control group. NSE-specific *Cre* transgenic mice (NSE-Cre mice) were obtained from Shanghai Biomodel Organism Science and Technology Development Co, Ltd. Mouse GFAP-specific *Cre* transgenic mice (GFAP-Cre mice; Stock No. 024098) were purchased from the Jackson Laboratory. Conditional GPR30–KO mice were generated by crossing GPR30*^fl/fl^* mice with GFAP-Cre or NSE-Cre mice, which resulted in the generation of GPR30*^fl/fl^*;GFAP-Cre and GPR30*^fl/fl^*;NSE-Cre mice. For transgenic mice, behavioral tests were performed during the postnatal tenth week. In all experiments, the operators were blinded to the grouping information.

### Ovariohysterectomy and drug treatment.

Eight-week-old female mice underwent ovariectomy under anesthesia with an oxygen/isoflurane mixture. Briefly, a dorsal incision was made, and the ovaries were removed. G1 (no. 10008933, Cayman) was dissolved in olive oil to a concentration of 2, 20, or 200 ng/μl, and 100 μl of G1 solution was s.c. injected into the necks of the mice once daily from the seventh day after surgery. For AG-KO female mice treatment, 100 μl of G1 (200 ng/μl) was s.c. injected to 9-week-old mice for 7 days, followed by behavioral tests.

### Virus construction and stereotactic injection.

Target genes and EGFP were coexpressed under the control of the GfaABC1D promoter. AAV2/5-GfaABC1D-EGFP, AAV2/5-GfaABC1D-Cre-EGFP, and AAV2/5-GfaABC1D-Cre were obtained from Obio Technology Corp. To induce GPR30 and PJA1 expression, AAVs were generated from the AAV-GfaABC1D-EGFP-P2A-*Gper1* (or *Pja1*)-3xFlag plasmid by Obio. The full-length cDNAs of *Gper1* and *Pja1* were transcribed from a mouse cDNA library. For Serpina3n knockdown, engineered AAVs carrying *Serpina3n*-shRNA (sequence: 5′-CTGATAATGATCTTTGACA-3′) or negative control (sequence: 5′-CTCGCTTGGGCGAGAGTAAG-3′) were also produced by Obio. AAV2/5-GfaABC1D-GCaMP6f was purchased from Brain Case (BC-0378).

The mice were anesthetized with gaseous isoflurane. AAVs were injected into the CA1 area (–1.7 mm anteroposterior, –1.7 mm mediolateral, and –1.6 mm dorsoventral) under stereotaxic guidance. For the injection of single AAVs, viruses (1 μl/site) were microinjected into the CA1 region using a Hamilton syringe (10 μl) connected to a 34-gauge metal needle after a hole was drilled in the skull. For the injection of 2 viruses, the viruses were mixed at a 1:1 ratio and coinjected into the CA1 region in a volume of 2 μl/site. The wounds were disinfected with 0.5% iodophor to prevent infection after suturing.

### Cell culture and drug treatment.

Primary astrocytes were prepared as previously described (60). Briefly, the cerebral tissues of 3-day-old mice (C57BL/6J) were digested with 0.125% trypsin/0.05% EDTA and dissociated into single cells. The cells were seeded in 75 cm^2^ polylysine-precoated dishes containing DMEM supplemented with 10% FBS and cultured at 37°C in an incubator containing 95% air and 5% CO_2_. When the cell confluence reached 90%, the dishes were rotated at 260 rpm (24 hours, 37°C) for the collection of purified astrocytes. Third-generation astrocytes were used. NHAs (BFN60808805, BLUEFBIO; provided by Wei Zhang, State Key Laboratory of Cancer Biology, Biotechnology Center, School of Pharmacy, Fourth Military Medical University, Xi’an, China) derived from female donors and were cultured in DMEM supplemented with 10% FBS. The cells were subcultured every 5 days at a subculture ratio of 1:3.

G1, G15 (No.14673, Cayman), and H89 (HY-15979A, MCE) were dissolved in DMSO and diluted in culture medium (for a final concentration of DMSO under 0.05%). To test cAMP levels, mouse primary astrocytes were treated with different concentrations of G1 or 1 nM G1 for 0, 10, 20, and 30 minutes. In other cases, primary astrocytes and NHAs were pretreated with 100 nM G15 or 10 μM H89 for 15 minutes before 1 nM G1 treatment for 2 hours.

### NOR test.

NOR test was performed as described previously (61). Each mouse was habituated to the behavioral apparatus (30 × 30 × 30 cm) for 15 minutes in the absence of objects. 2 identical cones were placed in different corners of the box. The mice were allowed to freely explore these 2 objects for 10 minutes (training trial). An hour later, 1 of the cones was replaced with a novel cylinder with a similar bottom area and height. The times spent exploring the novel (TN) and the familiar (TF) objects were recorded during a 5-minute reexploration trial (Test 1). Interaction was defined as contact with the object (excluding the tail) or facing the object (distance < 2 cm). After another 24 hour interval, a 5-minute retention trial in which the cylinder was replaced by a novel cube was performed (Test 2). The discrimination index was calculated as (TN − TF)/(TN + TF). Video-tracking and analysis were performed with DigBehv-LR4 software (Jiliang).

### FC test.

The FC apparatus consisted of a conditioning box (25 × 25 × 45 cm) with a grid floor connected to a shock generator and controlled by DigBehv-LR4 software (Jiliang) surrounded by an acoustic chamber. The mice were placed in the conditioning box for 3 minutes, and then a pure tone (75 dB) was delivered for 15 seconds, followed by a 1 second foot shock (0.75 mA). This procedure was then repeated 5 times at 60-second intertrial intervals (training period). After 24 hours, contextual memory was tested in the original conditioning box for 5 minutes. In the cue memory test, the mice were put into a new chamber with altered contextual elements (floor, wall, and odor) 30 hours after conditioning. The cue memory test consisted of 3 minutes of habituation and 3 minutes of cue exposure (75 dB pure tone). Freezing responses were assessed during the conditioning and testing procedures.

### Multichannel field potential recordings.

Coronal slices (300 μm) containing the hippocampus were prepared with a vibratome (Leica VT1200S) in ice-cold artificial cerebrospinal fluid (ACSF). One slice was transferred to the prepared MED64 probe and perfused with fresh oxygenated ACSF at room temperature, maintaining a flow rate of 2 mL/min. The afferent Schaffer collateral-commissural pathway from the CA3 region to the CA1 region was stimulated by 1 of 64 available planar microelectrodes. A theta-burst stimulation (TBS; 5 trains of bursts with 4 pulses at 100 Hz and 200 ms intervals, repeated 5 times at intervals of 10 seconds) protocol was applied to induce LTP. All multichannel electrophysiological data were acquired and analyzed by MED64 Mobius software. LTP strength was estimated by the fEPSP slope 180 minutes after TBS stimulus. Only the data from activated channels around the stimulus channel were used for fEPSP analysis.

### Whole-cell patch-clamp recording.

Sagittal slices (350 μm) containing the hippocampus were obtained as mentioned above. After recovery, the slices were transferred to a recording chamber on the stage of an Olympus microscope with infrared digital interference contrast optics for visualizing whole-cell patch-clamp recordings. sEPSCs were recorded from CA1 pyramidal neurons with an Axon 200B amplifier (Axon Instruments). The recording pipettes (3–5 MΩ) were filled with a solution containing 145 mM K-gluconate, 5 mM NaCl, 1 mM MgCl_2_, 0.2 mM EGTA, 10 mM HEPES, 2 mM Mg-ATP, and 0.1 mM Na_3_-GTP (all from Sigma-Aldrich) (pH adjusted to pH 7.2 with KOH; 290 mOsm). Neurons were clamped at −70 mV. Access resistance (5–30 MΩ) was monitored throughout the experiment. The data were discarded if the change in access resistance was over 15% during an experiment.

### Western blot analysis.

Equal amounts of protein (40 μg) were separated on SDS polyacrylamide gels and electrotransferred onto PVDF membranes (Millipore), which were probed with primary antibodies against β-actin (1:10,000, no. A5316, Sigma-Aldrich), Drebrin (1:2,000, no. 626523, Biorbyt), PJA1 (1:1,000, no. 629950, Biorbyt), Serpina3n (1:2,000, no. AF4709, R&D Systems), CREB (1:1,000, no. 9197, Cell Signaling Technology), p-CREB (1:1,000, no. 9198, Cell Signaling Technology), GPR30 (1:1,000, no. ab39742, Abcam), AACT (1:1,000, no. ab205198, Abcam), and GFAP (1:1,000, no. 3670, Cell Signaling Technology) overnight at 4°C. Subsequently, the membranes were incubated with HRP-conjugated secondary antibodies — including goat anti-mouse IgG-HRP (no. sc-2005, Santa Cruz), goat anti-rabbit IgG-HRP (no. sc-2004, Santa Cruz), and donkey anti-goat IgG-HRP (no. sc-2020, Santa Cruz) — and visualized using an enhanced chemiluminescence (ECL) system (Perkin Elmer). Band density was quantified with ImageJ software. The density of each band was normalized to the β-actin band density. The density ratio of the control group was set as 100%, and the density of the other groups is presented as a percentage of that of the control group.

### Golgi staining and spine morphology analysis.

Unfixed whole brains were immersed in Golgi-Cox solution (5% potassium dichromate, 5% mercuric chloride, and 5% potassium chromate [all from Sigma-Aldrich]) and stored at room temperature for 12 days. After dehydration in 70%, 90%, and 100% ethanol and ethanol/diethyl ether (1:1), coronal slices (120 μm) containing the hippocampus were cut with a vibratome. Next, the slices were incubated in 16% ammonia for 30 minutes. After washing in distilled water for 5 minutes, the sections were immersed in 1% sodium thiosulfate to fix the stain for 10 minutes. After gradient dehydration, the slices were hyalinized with xylene and mounted onto slides with neutral gum. Pictures were captured under a Nikon DS-5M-UI 80i light microscope with a 100×/NA 1.4 oil immersion lens. The density of secondary basal dendrites of CA1 neurons was analyzed with ImageJ software. Spine morphology was classified according to the following criteria: “thin” spines, which had long necks and small heads; “filopodia” spines, which had long processes but no heads; “stubby” spines, which had broader heads but no necks; and “mushroom” spines, which had thin necks and bulbous heads.

### Immunofluorescence staining.

Brains were carefully removed and postfixed in 4% paraformaldehyde overnight at 4°C. After dehydration, coronal sections (10 μm) containing the hippocampus were cut using a freezing microtome (CM1950, Leica). The sections were permeabilized with 0.3% Triton X-100 in normal goat serum for 1 hour. Then, the sections were incubated with GFAP antibody (1:400, no. GB11096, Servicebio) overnight at 4°C. After washing, the sections were incubated with Cy3-conjugated goat anti-rabbit secondary antibody (1:300; no. GB21303, Servicebio) for 1 hour at room temperature. DAPI was used to stain nuclei. All sections were mounted onto glass slides with cover glass using 50% glycerinum. For astrocyte morphology analysis, fluorescence images were acquired using a confocal laser-scanning microscope (Nikon ECLIPSE C1). A stack of images of each slice spanning a thickness of 5 μm were taken at an optical step of 1 μm. The freehand selection tool in ImageJ was employed to measure the cell body area. Sholl analysis (starting radius: 4 μm, ending radius: 50 μm, radius step size: 2 μm) was performed to analyze astrocyte branches and processes.

### qRT–PCR.

Total RNA was extracted by an RNAprep FastPure kit (TSP413, Tsingke) according to the manufacturer’s instructions. Goldenstar RT6 cDNA Synthesis Mix (TSK134S, Tsingke) was used to synthesize cDNA from the RNA. The reactions were performed using 2 × T5 Fast qPCR Mix (SYBR Green I) (TSE202, Tsingke). The sequences of the primers are listed in Supplemental Table 1. The *GAPDH* gene was used as a reference gene. Changes in gene expression are shown as fold changes compared with the gene expression level in the controls.

### RNA-Seq.

RNA-Seq was conducted by LC-Bio Technology Co, Ltd. Briefly, Poly (A) RNA was purified from 1 μg total RNA using Dynabeads Oligo (25-61005, Thermo). Then, the poly (A) RNA was fragmented into small pieces using a Magnesium RNA Fragmentation Module (m6150, NEB) at 94°C for 5 to 7 minutes. The cleaved RNA fragments were converted into cDNA by SuperScript II Reverse Transcriptase (1896649, Invitrogen). The DNA was purified from enzymatic reactions and the size selection of the library was performed with AMPureXP beads. After heat-labile UDG enzyme (m0280, NEB) treatment of the U-labeled second-stranded DNAs, the ligated products were amplified with PCR. The average insert size for the final cDNA library was 300 ± 50 bp. we performed 2 × 150 bp paired-end sequencing (PE150) on an Illumina NovaSeq 6000 platform (LC-Bio) following the vendor’s recommended protocol. HISAT2 (https://ccb.jhu.edu/software/hisat2) was used to map reads to the reference genome of *Mus musculus* GRCm38. After transcriptome generation, StringTie was used to estimate the levels of all transcripts by calculating the FPKM (FPKM = [total exon fragments/mapped reads (millions) × exon length (kB)]). mRNAs with a fold change greater than 2 or fold change less than 0.5 and *P* less than 0.05 were selected as differentially expressed mRNAs. The RNA-Seq data sets are available at the NCBI GEO website (accession GEO: GSE212893). GO enrichment analysis was performed using DAVID (https://david.ncifcrf.gov/).

### Co-IP.

Hippocampal or cell samples were lysed in NP-40 lysis buffer (HY-K0202A, MCE; P0013F, Beyotime) containing protease inhibitor. Equal amounts of protein were incubated with antibody or homologous IgG overnight at 4°C. Subsequent pull-down was performed by adding protein A/G magnetic agarose beads (78610, Thermo Fisher Scientific) for an additional 2 hours at 4°C. The beads were collected, washed 3 times with lysis buffer, and boiled in 2× SDS gel loading buffer. The samples were analyzed by Western blotting as described above.

### IP followed by MS.

IP was performed as described above. The sample was incubated with an anti-PJA1 antibody (#629950, Biorbyt). The beads were subjected to MS by Shanghai Bioprofile Biotechnology Co, Ltd. Briefly, the bound proteins were extracted from the IP beads using SDT lysis buffer (4% SDS, 100 mM DTT, 100 mM Tris-HCl pH 8.0). The sample was digested by the filter-aided sample preparation (FASP) method as described by Wisniewski, et al. (62). Liquid chromatography linked to tandem mass spectrometry (LC–MS/MS) was performed on a Q Exactive Plus mass spectrometer coupled to an Easy nLC instrument (Thermo Fisher Scientific). The MS data were analyzed using MaxQuant software version 1.6.0.16 and searched against the UniProtKB *Mus musculus* database (8,8061 total entries, downloaded 04/16/2021). The maximal 2 missed cleavage sites and the mass tolerance of 4.5 ppm for precursor ions and 20 ppm for fragment ions were defined for the database search. Carbamidomethylation of cysteines was defined as a fixed modification, while acetylation of the N-terminal protein and oxidation of methionine were set as variable modifications for the database search. Database search results with a less-than 1% FDR at the peptidespectrum-matched level and protein level were filtered and exported.

### Prediction of the CREB binding sites in the promoter of Pja1.

The mouse and human *Pja1* promoter sequences were obtained from the UCSC database (http://genome.ucsc.edu/). The region from 2,000 bp upstream and 100 bp downstream of the transcriptional start site was considered the promoter region. The transcript IDs of mouse and human *Pja1* were mm39_knownGene_ENSMUST00000167246.2 and hg38_knownGene_ENST00000361478.1, respectively. The CREB binding motif was acquired from the JASPAR database (http://jaspar.genereg.net/). The promoter region of Pja1 was input into the JASPAR database to predict the potential CREB-binding sites, which were ranked according to their score. The top 3 binding sites were selected for validation by ChIP.

### ChIP.

ChIP was performed using a kit from BersinBio (Bes5001) according to the manufacturer’s instructions. DNA–protein crosslinking was performed by fixing the cells with 1% formaldehyde at room temperature for 10 minutes. The cross-linked chromatin was sonicated in lysis buffer from the kit. DNA fragments were obtained by centrifugation at 20,000*g* for 10 minutes. The supernatants were used for IP. The anti-CREB or rabbit IgG-coupled protein A/G beads were added and incubated for 4 hours at 4°C. The beads were washed twice with wash buffer. Then, the DNA was eluted in elution buffer, decrosslinked at 65°C overnight, purified via phenol/chloroform/isoamyl alcohol extraction and ethanol precipitation, and subjected to qRT–PCR. The primers are listed in Supplemental Table 1. After amplification, the PCR products were run on a 2.5% agarose gel containing ethidium bromide.

### ELISA.

The levels of cAMP, estrogen, and AACT were measured with ELISA kits (KGE002B, R&D Systems; ZC-32658, ZCIBIO; JL13071, Jianglaibio) following the manufacturer’s instructions. The human plasma was directly used to detect estrogen and AACT. Astrocytes were lysed in Cell Lysis Buffer by repeated freeze/thaw cycles. After brief centrifugation (600g for 10 minutes), the supernatant was used for ELISA. The levels of cAMP cAMP, estrogen, and AACT were quantified by Ascent Software for Multiskan.

### Statistics.

All statistical analyses were performed by operators blinded to the experimental conditions and conducted using GraphPad Prism software (version 7.02) or SPSS 19.0. All data are expressed as the mean ± SEM. We assessed the significance of differences between 2 groups by 2-tailed independent sample *t* tests. 1-way ANOVA followed by Tukey’s posthoc test was used to compare means among multiple groups. Repeated-measures 2-way ANOVA followed by the Tukey’s posthoc test was used to compare means when there were 2 variables. All comparisons are 2-tailed. In all cases, *P* < 0.05 was considered statistically significant.

### Study approval.

The animal experimental procedures were approved by The Animal Care and Use Committee of the Fourth Military Medical University (20200460). Women’s blood samples were obtained from The Second Affiliated Hospital of Fourth Military Medical University. The study was approved by the Ethics Committee of The Second Affiliated Hospital of Air Force Medical University (202210-03), and written informed consent was obtained from all subjects.

### Data availability.

Values for all data points in graphs are reported in the Supporting Data Values file. The RNA-Seq data sets are available from the NCBI GEO website (accession GEO: GSE212893). 

## Author contributions

SL, MZ, and XW designed the experiments, wrote the manuscript, and secured funding. XW performed or participated in all experiments described in the manuscript. YJ, KZ, LY, JY, and LL performed surgery, viral injection, behavior experiments, and Western blot analysis. DS, JQ, XL, and MW contributed to the confocal imaging, astrocyte morphological analysis, and ChIP. BF, QG, and LY performed all slice electrophysiology, LTP, and fiber photometry recording. FY and LL contributed to cell culture. XM and ZL supervised the study and contributed to bioinformatics analysis. HZ and JH contributed to recruitment of volunteers for clinical samples. All authors have agreed on the final version to be published.

## Supplementary Material

Supplemental data

Supplemental video 1

Supporting data values

## Version 1 09/15/2023

Electronic publication
