# Supplementary material for: PJA1 mediates the effects of astrocytic GPR30 on learning and memory in female mice
Source: J Clin Invest. 2023 Sep 15;133(18):e165812. doi: 10.1172/JCI165812 (PMC10503807; doi:10.1172/JCI165812)
Supplement: Supplemental data [file jci-133-165812-s058.pdf]

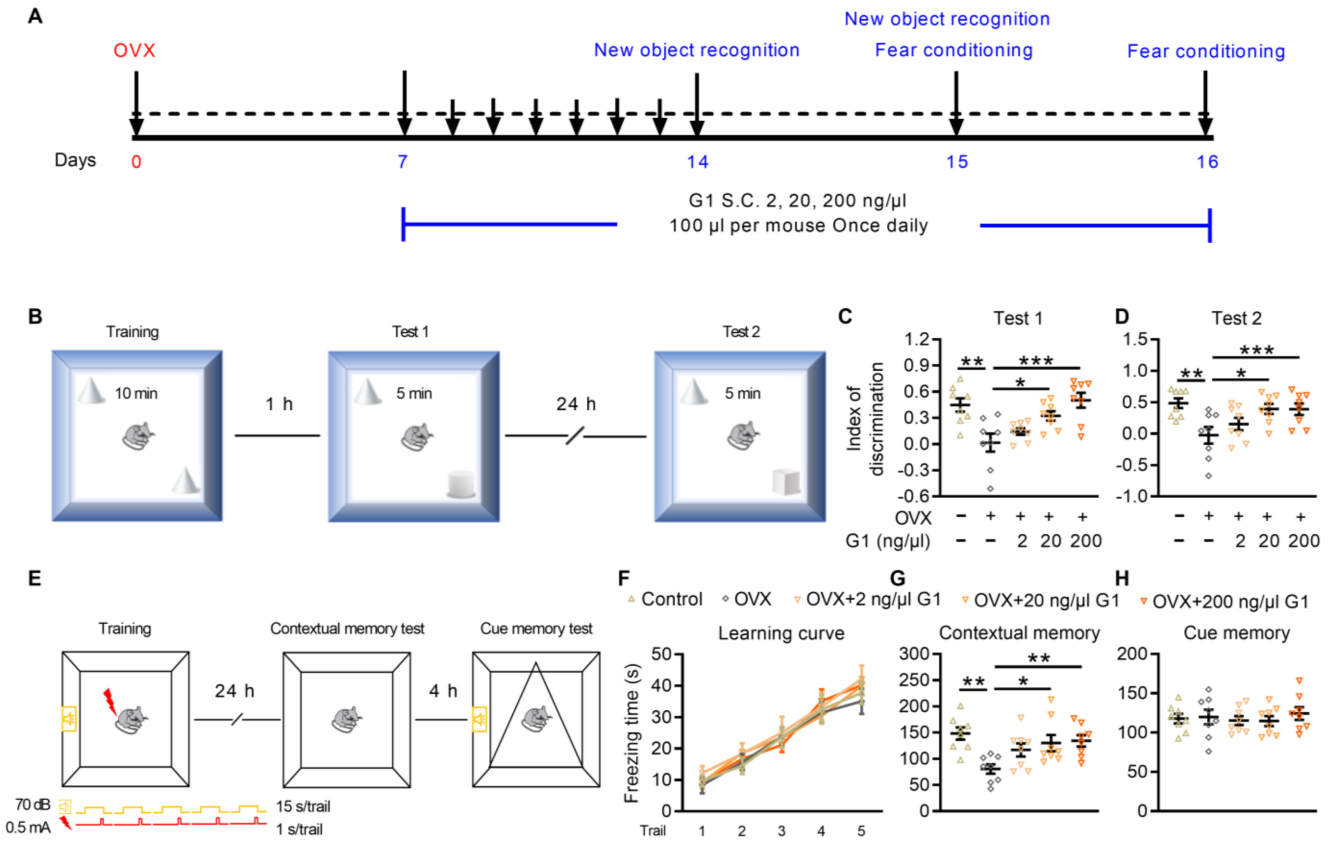

Supplemental Figure 1. GPR30 agonist G1 rescues memory impairment in OVX mice

(A) Experimental protocol. (B-D) Schematic of the NOR test (B). G1 treatment increased the discrimination index of OVX mice in a dose-dependent manner in Test 1 (C) and Test 2 (D). *n* = 8 mice per group. (E-H) Schematic of the FC test (E). Freezing time was recorded, and there was no difference in freezing time in the training phase (F) or the cue memory test (H). The freezing time in the contextual memory test was increased after G1 treatment (G). *n* = 8 mice per group. Data are presented as mean ± SEM. *$P < 0.05$, **$P < 0.01$, ***$P < 0.001$ by one-way ANOVA with Tukey's post hoc test (C/D/G/H) or two-way ANOVA with Tukey's post hoc test (F).

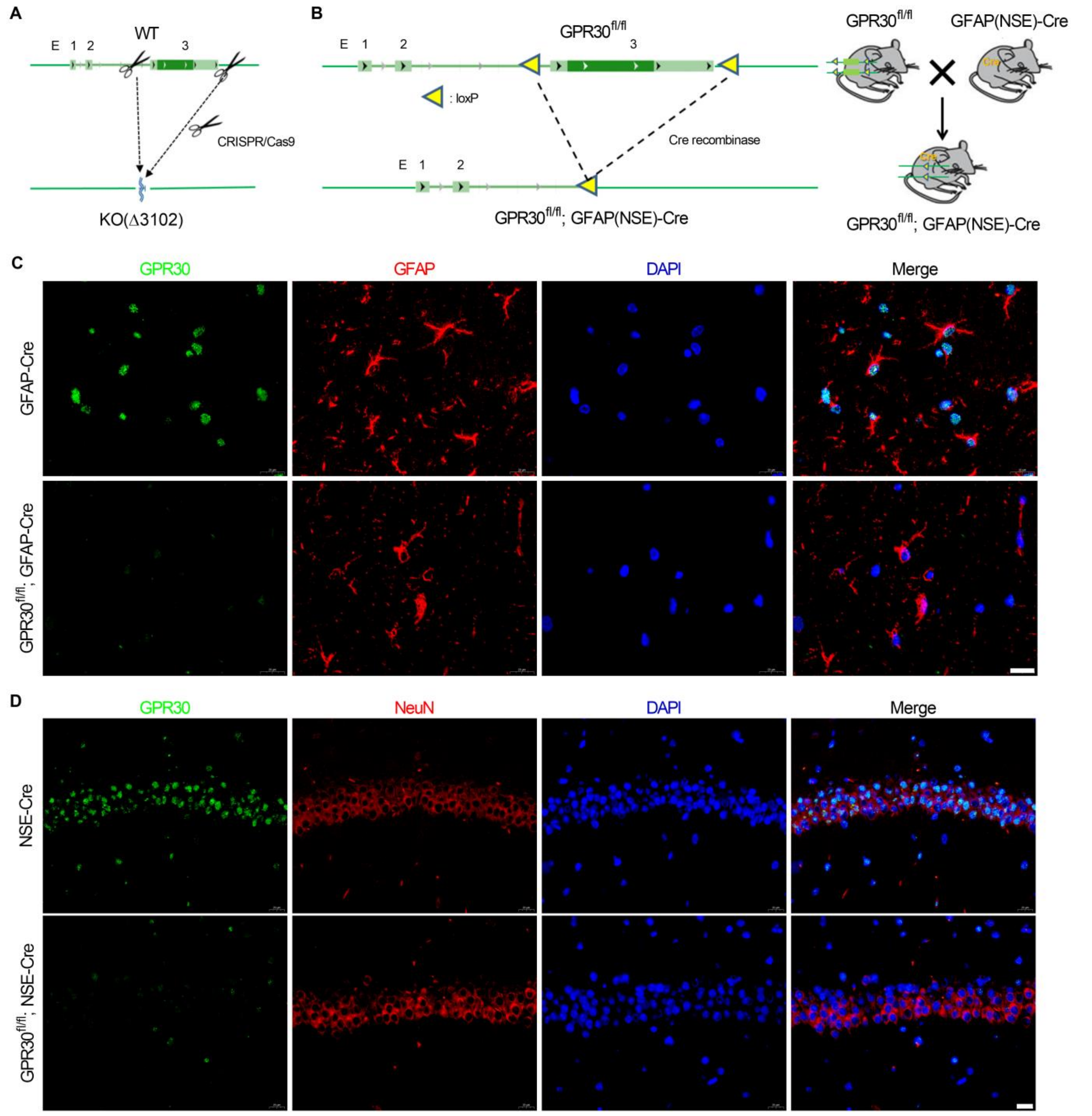

Supplemental Figure 2. Identification of *GPR30* mRNA deletion in conditional KO mice

(A and B) Schematic of global GPR30 KO (Δ3102) and lineage-specific KO mouse generation. (C) GFAP immunostaining was performed after FISH for *GPR30* probes. Scale bar: 20 μm. (D) NeuN immunostaining was performed after FISH for *GPR30* probes. Scale bar: 20 μm. Female mice were used in the experiment.

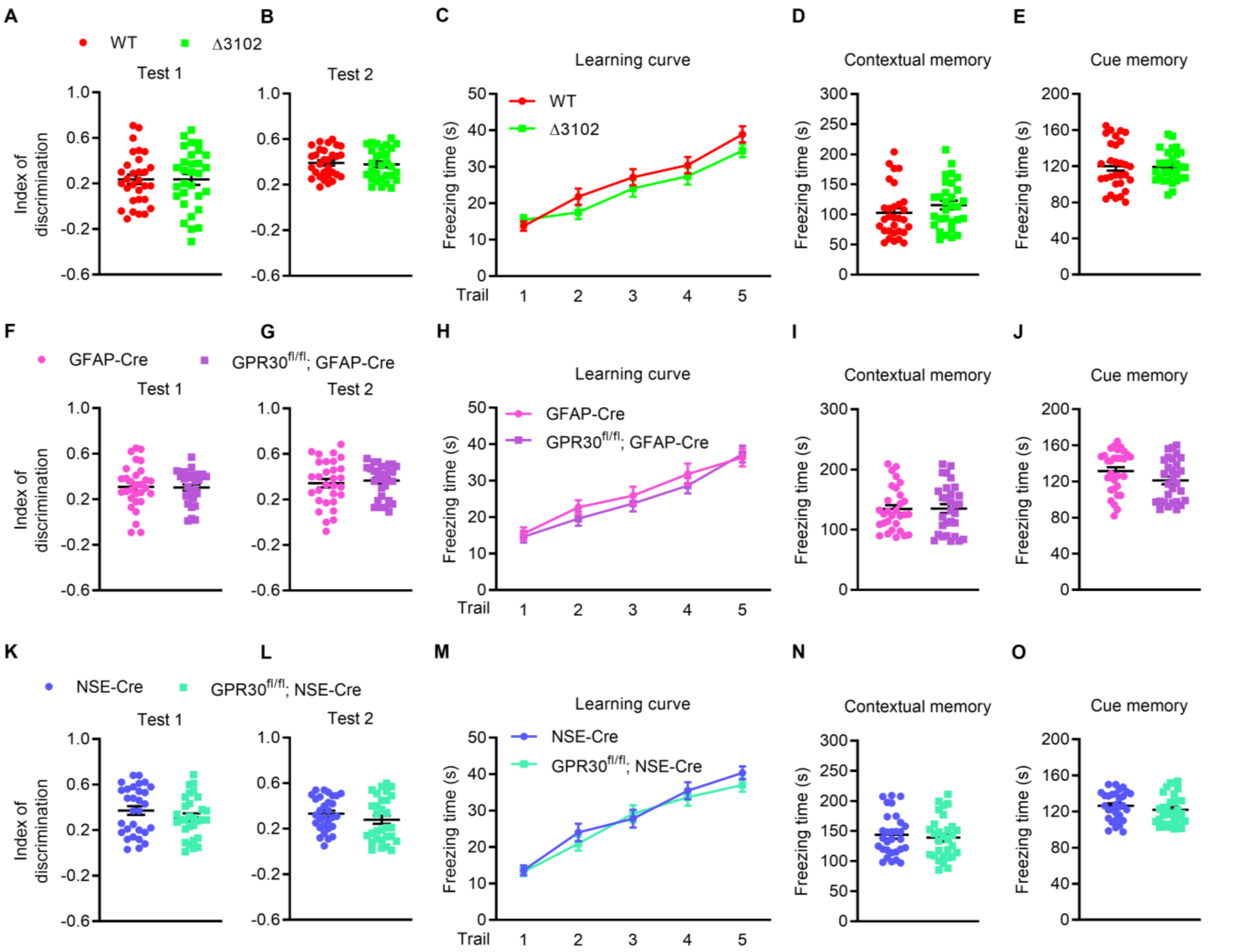

Supplemental Figure 3. Learning and memory are not altered in male GPR30 transgenic mice

(A-E) The discrimination index in the NOR test (A and B) and freezing time in the FC test (C-E) were not changed in male Δ3102 mice. *n* = 30 mice per group. (F-J) AG-KO male mice showed a normal discrimination index in the NOR test (F and G) and freezing time in the FC test (H-J). *n* = 30 mice per group. (K-O) There was no difference in the discrimination index in the NOR test (K and L) or freezing time in the FC test (M-O) between NG-KO and male control mice. *n* = 30 mice per group. Δ3102: GRR30 KO. AG-KO: $GPR30^{fl/fl}$;GFAP-Cre. NG-KO: $GPR30^{fl/fl}$;NSE-Cre. WT, GFAP-Cre, and NSE-Cre are control mice. Data are presented as mean ± SEM. *P* values were caculated by independent sample t test (A/B/D/E/F/G/I/J/K/L/N/O) or two-way ANOVA (C/H/M)

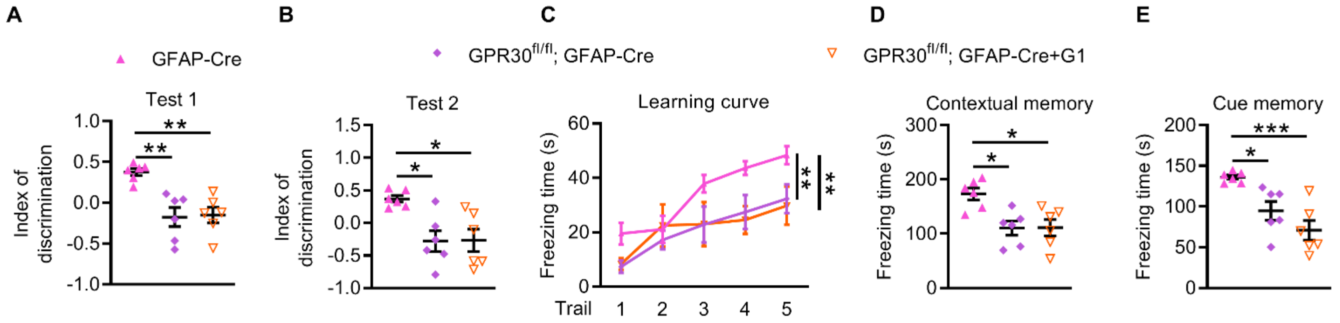

Supplemental Figure 4. GRP30 agonist G1 has no effect on learning and memory impairment in AG-KO female mice

(A-D) G1 treatment didn't reverse the decrease of discrimination index (A and B) and freezing time (C-E) in AG-KO female mice. $n = 6$ female mice per group. AG-KO: GPR30$^{fl/fl}$;GFAP-Cre. GFAP-Cre: control mice. Data are presented as mean ± SEM. *$P < 0.05$, **$P < 0.01$, ***$P < 0.001$ by one-way ANOVA with Tukey's post hoc test (A/B/D/E) or two-way ANOVA with Tukey's post hoc test (C).

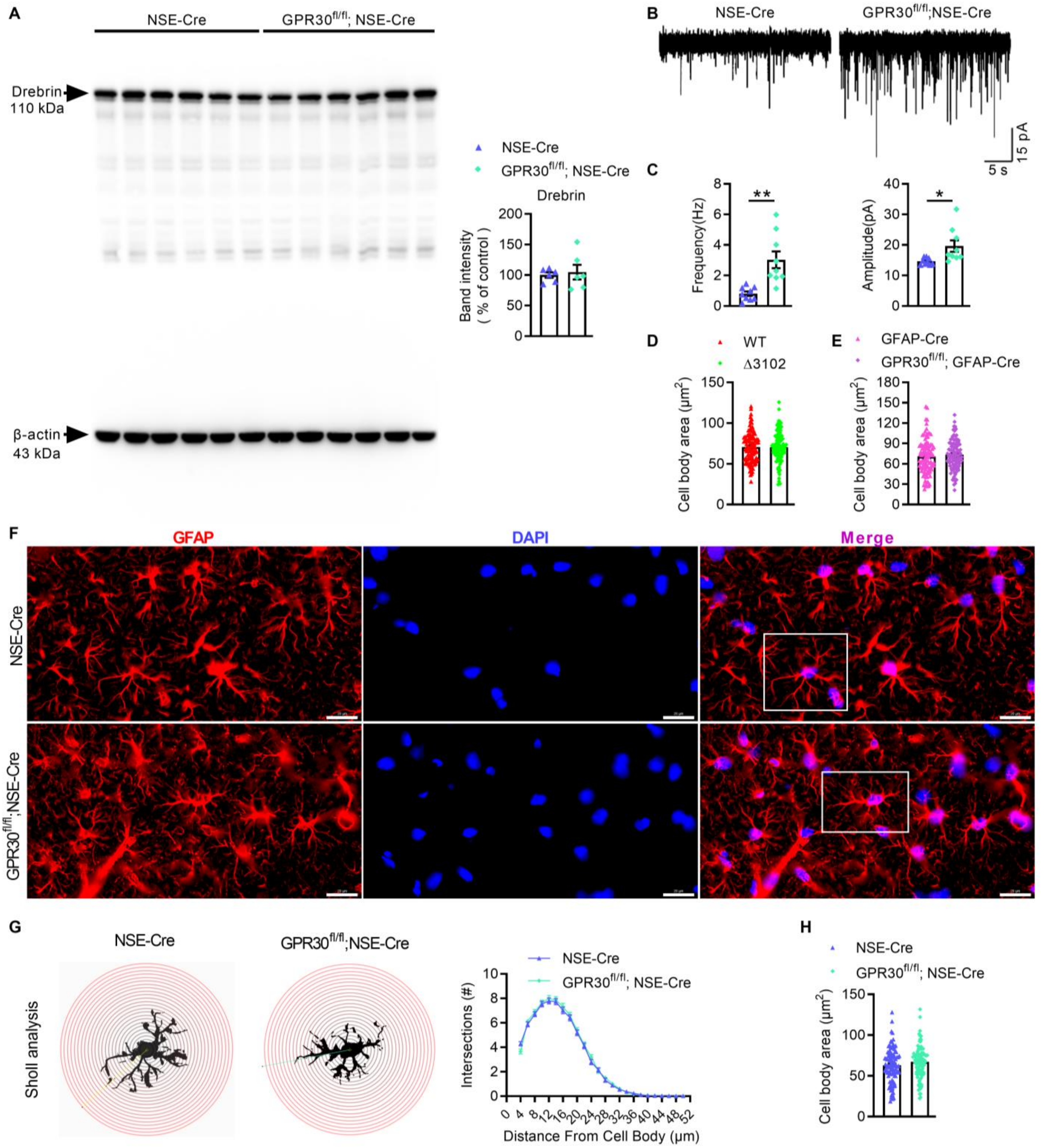

Supplemental Figure 5. Neuronal and astrocytic changes in the hippocampi of NG-KO female mice

(A) Immunoblots of Drebrin in hippocampal protein lysates from NG-KO female mice. *n* = 6 mice per group. (B and C) Representative traces of sEPSCs in CA1 hippocampal pyramidal neurons from control and female NG-KO mice (B). Both the frequency and amplitude of sEPSCs were increased in NG-KO female mice (C). *n* = 9 neurons from 3 mice per group. (D and E) Quantification of the astrocytic cell body area in Δ3102 (D) and AG-KO mice (E). *n* = 100 cells from 5 mice per group. (F and G) Confocal images of GFAP staining (F) and Sholl analysis of astrocyte complexity in hippocampal slices from NG-KO female mice (G). Scale bar: 20 μm. *n* = 100 cells from 5 mice per group. (H) Quantification of the astrocytic cell body area in NG-KO female mice. *n* = 100 cells from 5 mice per group. Δ3102: GRR30 KO. AG-KO: $GPR30^{fl/fl}$;GFAP-Cre. NG-KO: $GPR30^{fl/fl}$;NSE-Cre. WT, GFAP-Cre, and NSE-Cre are control mice. Data are presented as mean ± SEM. *$P < 0.05$, **$P < 0.01$ by independent sample t test (A/C/D/E/H) or two-way ANOVA (G).

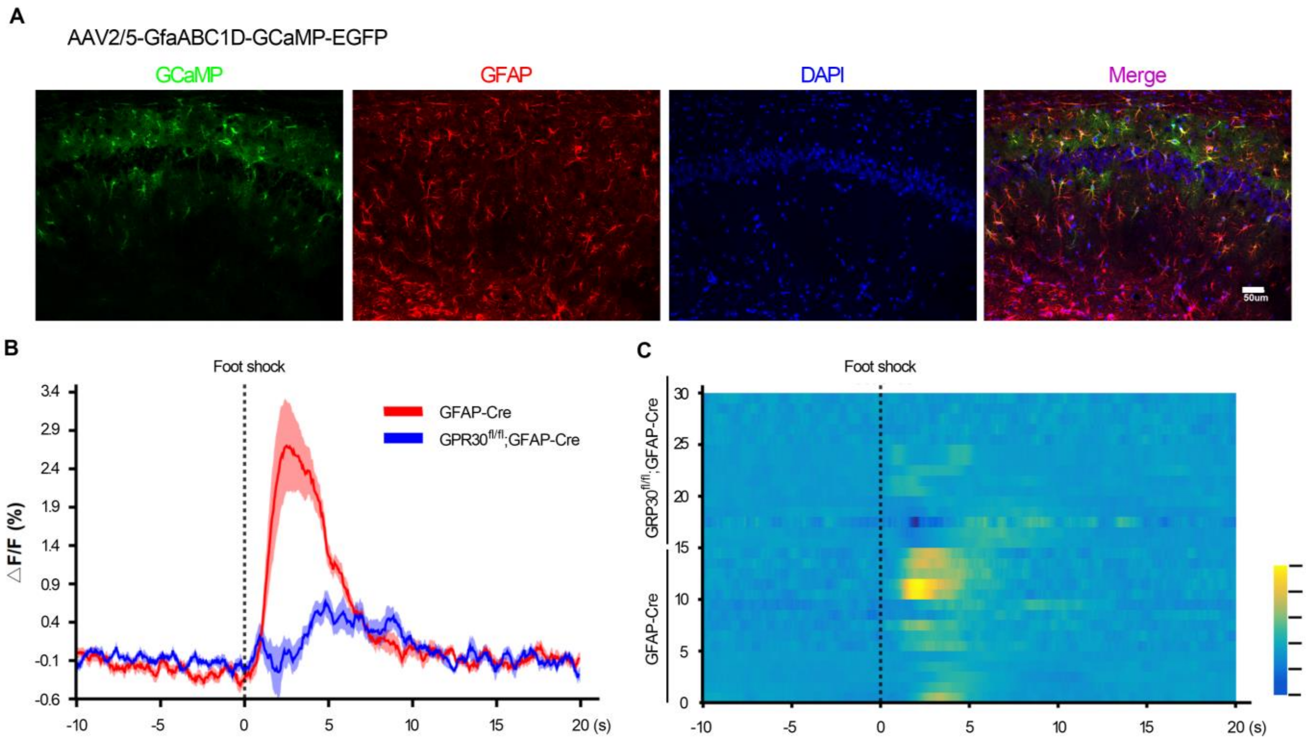

Supplemental Figure 6. Ca^{2+} activity in hippocampal astrocytes of AG-KO female mice

(A) Representative images showing the specific expression of GCaMP in astrocytes of the CA1 region. Scale bar: 50 μm. (B and C) Mean Ca^{2+} signal intensity (B) and heatmap (C). The results showed that the Ca^{2+} signal intensity was much lower in AG-KO female mice than in control mice upon exposure to foot shock. The colored bars indicate ΔF/F (%). AG-KO: $GPR30^{fl/fl}$;GFAP-Cre. GFAP-Cre: control mice. Data are presented as mean ± SEM.

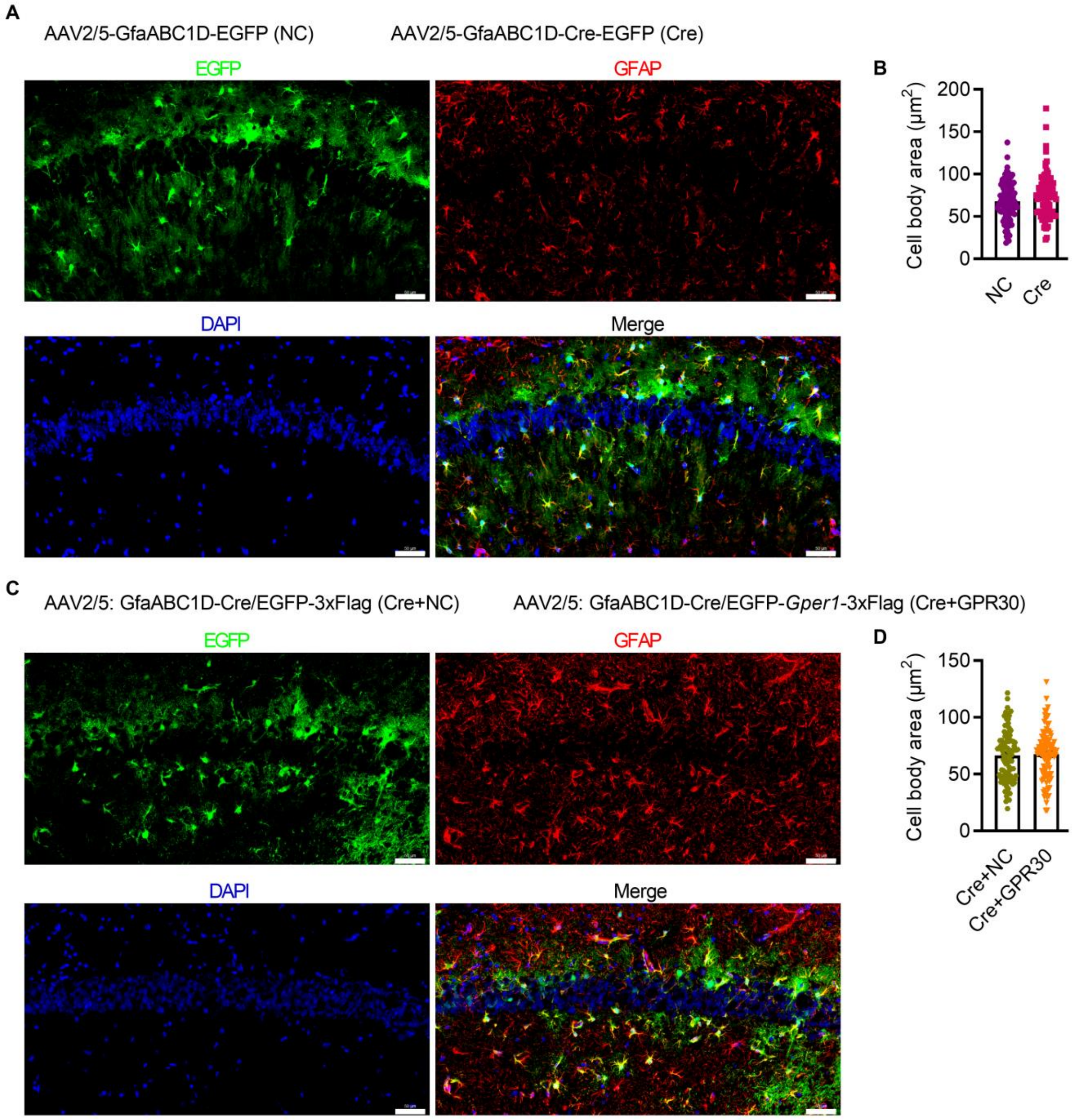

Supplemental Figure 7. Specific expression of AAV viruses in astrocytes and quantification of the astrocytic cell body area

(A) Representative images showing the specific expression of AAV viruses in astrocytes, which induced GPR30 deletion in astrocytes. Scale bar: 50 μm. (B) Cre-induced GPR30 deletion in astrocytes had no effect on the astrocytic cell body area. n = 100 cells from 3 mice per group. (C) Representative images confirming the expression of the viruses in astrocytes, which induced GPR30 re-expression after GPR30 deletion in astrocytes. Scale bar: 50 μm. (D) Restoration of GPR30 expression in astrocytes did not influence the astrocytic cell body area. n = 100 cells from 3 mice per group. NC indicates control mice. Cre/Cre+NC indicates astrocytic GPR30 KO mice. Cre+GPR30 indicates mice with astrocytic GPR30 KO and restoration of GPR30 expression. Data are presented as mean ± SEM. *P* values were caculated by independent sample t test.

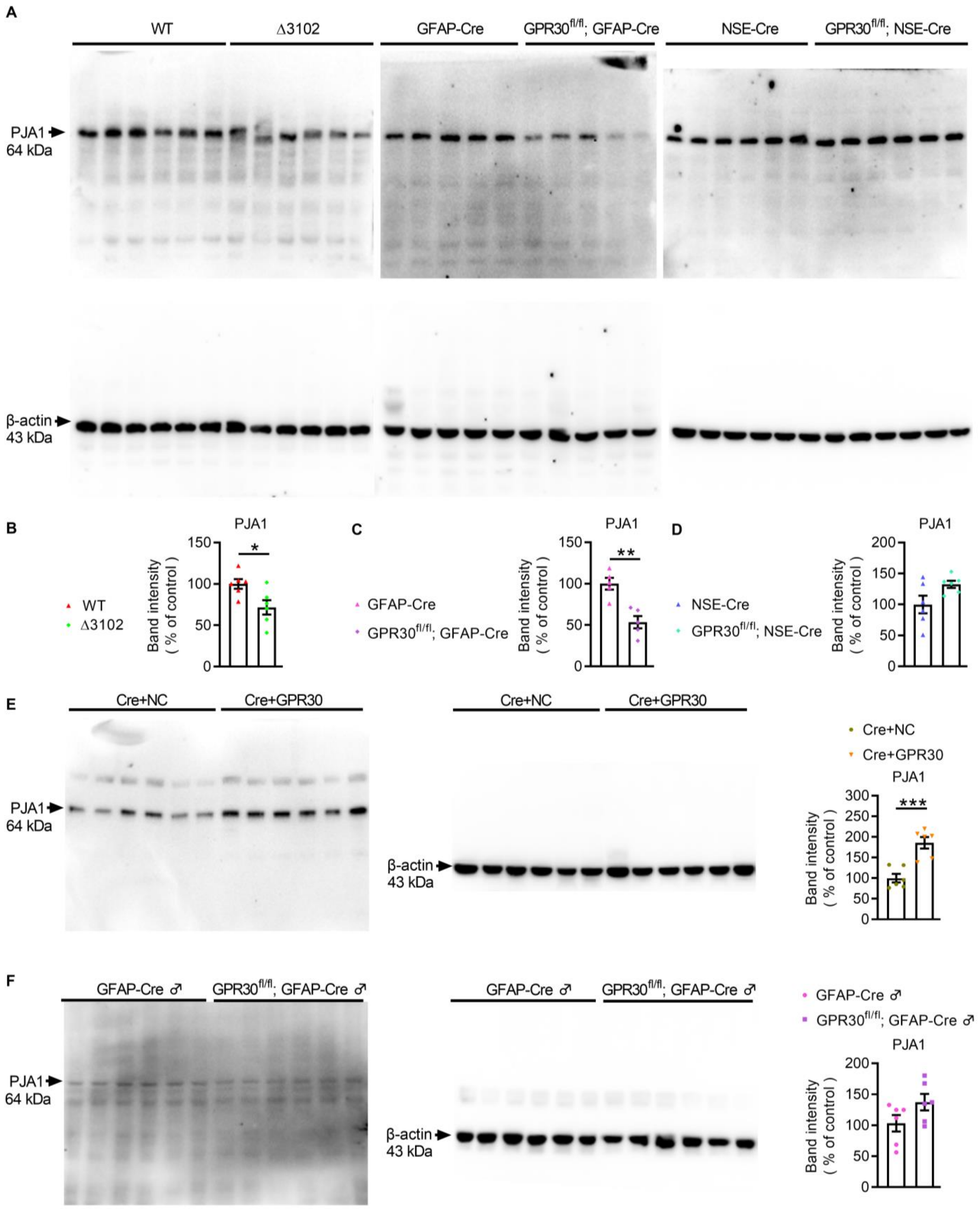

Supplemental Figure 8. PJA1 levels in the hippocampi of female and male transgenic mice

(A-D) Immunoblots of PJA1 in the hippocampi of Δ3102, AG-KO, and NG-KO female mice. The levels of PJA1 were significantly decreased in Δ3102 (B, *n* = 6 mice per group) and AG-KO mice (C, *n* = 5 mice per group) but unchanged in NG-KO mice (D, *n* = 6 mice per group). Δ3102: GRR30 KO. AG-KO: $GPR30^{fl/fl}$;GFAP-Cre. NG-KO: $GPR30^{fl/fl}$;NSE-Cre. WT, GFAP-Cre, and NSE-Cre are control mice. (E) Immunoblots of PJA1 in the hippocampi of control and Cre+GPR30 female mice. Restoration of GPR30 expression in astrocytes increased PJA1 levels. *n* = 6 mice per group. (F) Immunoblots of PJA1 in the hippocampi of AG-KO male mice, and PJA1 was unchanged in AG-KO male mice. *n* = 6 mice per group.

Cre+NC indicates astrocytic GPR30 KO mice. Cre+GPR30 indicates mice with astrocytic GPR30 KO and restoration of GPR30 expression. Data are presented as mean ± SEM. *$P < 0.05$, **$P < 0.01$, ***$P < 0.001$ by independent sample t test.

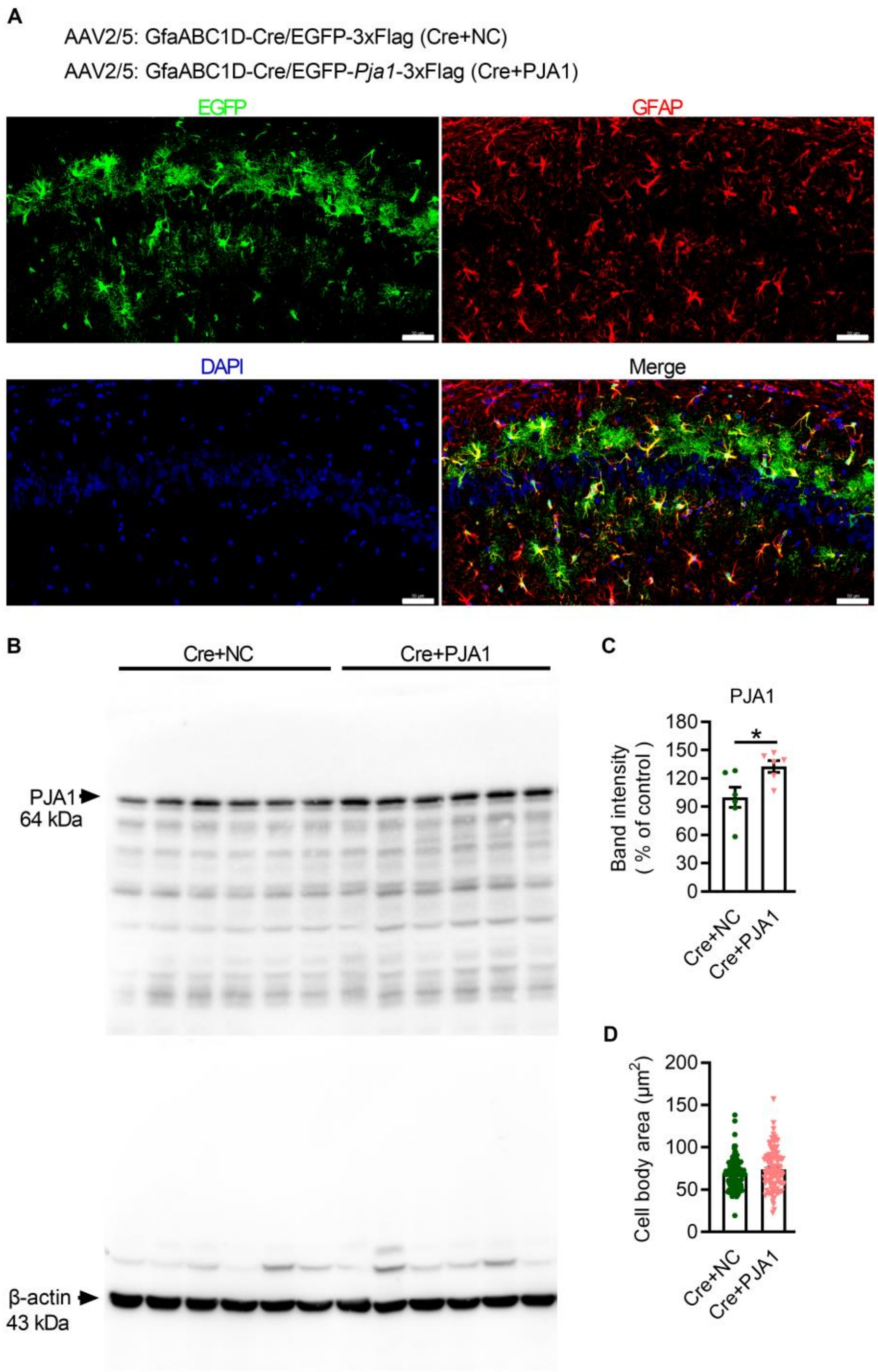

Supplemental Figure 9. Specific upregulation of PJA1 in astrocytes and the quantification of astrocytic cell body area

(A) Representative images showing the specific expression of PJA1-expressing viruses in astrocytes of the CA1 region. Scale bar: 50 μm. (B and C) Immunoblots of PJA1 in the hippocampi of control and Cre+PJA1 mice (B). PJA1 was upregulated after PJA1-expressing AAV injection (C). n = 6 mice per group. (D) The cell body area had no change after PJA upregulation in astrocytes. n = 100 cells from 3 mice per group. Cre+NC indicates astrocytic GPR30 KO mice. Cre+PJA1 indicates astrocytic GPR30 KO and PJA1 upregulation mice. Data are presented as mean ± SEM. *P < 0.05 by independent sample t test.

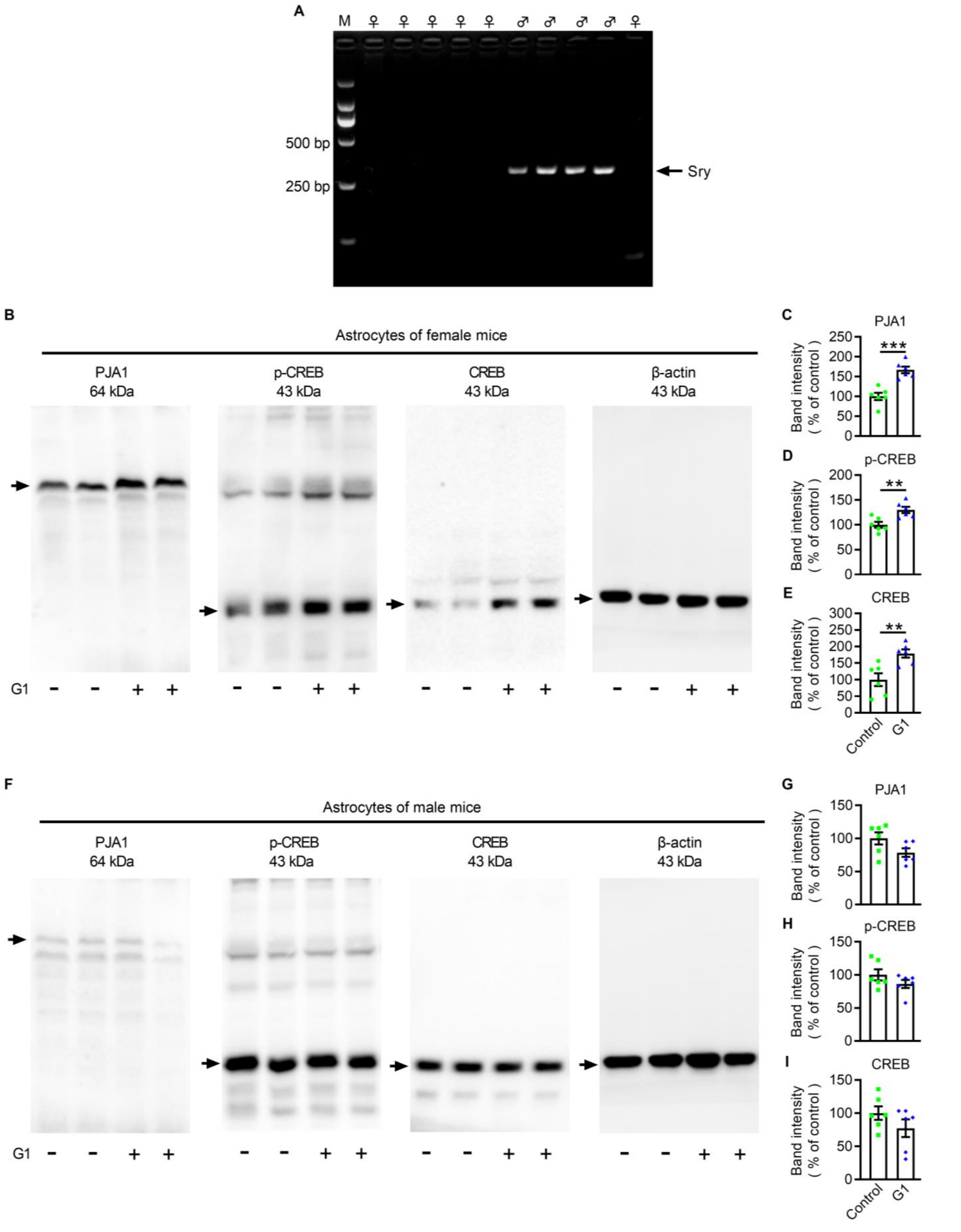

Supplemental Figure 10. GPR30 regulates PJA1 only in female astrocytes

(A) Sry gene was detected to determine the sex of newborn mice. (B-E) Representative immunoblots and quantification of PJA1 (C), p-CREB (D), and CREB (E) in cultured female astrocytes treated with G1. $n = 6$ samples per group. (F-I) Representative immunoblots and quantification of PJA1 (G), p-CREB (H), and CREB (I) in cultured male astrocytes treated with G1. $n = 6$ samples per group. Data are presented as mean ± SEM. $^{**}P < 0.01$, $^{***}P < 0.001$ by independent sample t test.

Sequence: Pja1 promoter sequence.dna (Linear / 2000 bp)
Features: 3 visible, 3 total
Primers: 6 visible, 6 total

```
5' ATGTTAAGGTAAACTAGGTAAGGCAAAGCTTCGGGTGCAATTTTCTTGTTTTAGAAGTAGAGGCTCTGAAGTTTGAACAA   80
3' TACAATTCCATTTGATCCATTCCGTTTCGAAGCCCACGTTAAAAGAACAAAATCTTCATCTCCGAGACTTCAAACTTGTT

   AAGCAAAAGTTGCTCTGTGTGACGGATGAATTCCAATAGGTATTTTACACTGTATGTTTTCTTGTTTGAAATGAGTATTT   160
   TTCGTTTTCAACGAGACACACTGCCTACTTAAGGTTATCCATAAAATGTGACATACAAAAGAACAAACTTTACTCATAAA

   TTATAAAACAAAACAAAACCCTGAATCCTCCTGAGTAGAAAAAATGCATATGTGTTAGCTCCAACTTATTAACTGTTGTT   240
   AATATTTTGTTTTGTTTTGGGACTTAGGAGGACTCATCTTTTTTACGTATACACAATCGAGGTTGAATAATTGACAACAA

   CAGTCTCCCCGTGTCTTTCCTCATTTAGAAGTCCATTTGATATGTCTATAGCACCACTACTACTATACTACTTCCTGACG   320
   GTCAGAGGGGCACAGAAAGGAGTAAATCTTCAGGTAAACTATACAGATATCGTGGTGATGATGATATGATGAAGGACTGC

   GCTGCGTATGTTTTGAAATTCCATCATTTGACCCATACACTTTATGATTATTCTGCCTCTTTTGGTTTCTGAAATGACTA   400
   CGACGCATACAAAACTTTAAGGTAGTAAACTGGGTATGTGAAATACTAATAAGACGGAGAAAACCAAAGACTTTACTGAT

   TTGTGTTCTCTTCAGCACTTTTTACTTTGACTTATTTACTTGTAGATTAGTATTGCTAATTTGAATGTGCAAGCTCCAGC   480
   AACACAAGAGAAGTCGTGAAAAATGAAACTGAATAAATGAACATCTAATCATAACGATTAAACTTACACGTTCGAGGTCG

   AACCTAGAAAGTTCCAGGCGAGCCTGAGGTCCATGAGAGCTTGTCTGACAATAACTGCAACAAACCCTCCTGACACCCTA   560
   TTGGATCTTTCAAGGTCCGCTCGGACTCCAGGTACTCTCGAACAGACTGTTATTGACGTTGTTTGGGAGGACTGTGGGAT

   CTCCACCCCCTCCACTCCAACAAAGGAAAAAATGCTTTTTTTTTCTTCTTGAGACAGAGTCTTCCCTCTTTGTAACCCTG   640
   GAGGTGGGGGAGGTGAGGTTGTTTCCTTTTTTACGAAAAAAAAAGAAGAACTCTGTCTCAGAAGGGAGAAACATTGGGAC

   GCTATCCTGGAAGTCTCTATGTAGACCAGGCTGACCACAAAAAACAAACAGAAGCATACAAAACAAACAAACAAATGAAC   720
   CGATAGGACCTTCAGAGATACATCTGGTCCGACTGGTGTTTTTTGTTTGTCTTCGTATGTTTTGTTTGTTTGTTTACTTG

   AAAAACAAACCAGAAGTTCTTCTGCCTCTGCCTCCTCTGCCACCGCTGCCCCTGCCCCTGCCCCTGCCCCTGCCCCTGCC   800
   TTTTTGTTTGGTCTTCAAGAAGACGGAGACGGAGGAGACGGTGGCGACGGGGACGGGGACGGGGACGGGGACGGGGACGG

                                                                           Site 2 F
                                                                           GCAGAGCTG
   CCTGCCCCTGCCTCTCTGCCTCATTGCCTCTGCCTCTGCCTCTGCCTCTCTGCCTCATTGCCTCTGCCTCTGCAGAGCTG   880
   GGACGGGGACGGAGAGACGGAGTAACGGAGACGGAGACGGAGACGGAGAGACGGAGTAACGGAGACGGAGACGTCTCGAC

   Site 2 F
   GGATCAAAGAC
   GGATCAAAGACGTGAGCCAACATGTAGGCTGAATTTGGTTTTATGTAGATAGTAGTTCGTAGTACACACTGCCACGTGGG   960
   CCTAGTTTCTGCACTCGGTTGTACATCCGACTTAAACCAAAATACATCTATCATCAAGCATCATGTGTGACGGTGCACCC
        CREB binding site 2

   GATTGGCTCCTGCTTGGTTTCTTTCTCTACTCTTTGTTCTCGCCCACCCTCTCCTTGTTTCTTGTCCCCTTGTTCGATAT   1040
   CTAACCGAGGACGAACCAAAGAAAGAGATGAGAAACAAGAGCGGGTGGGAGAGGAACAAAGAACAGGGGAACAAGCTATA
                                                                  ACAGGGGAACAAGCTATA
                                                                            Site 2 R

   CCCCCCCCCCCGCTTAGATTTCCATAGTATTCTTGTAATAGAAATTGCCCATGCTGGAACTCTCCATTGAGTGTTCTAAG   1120
   GGGGGGGGGGGCGAATCTAAAGGTATCATAAGAACATTATCTTTAACGGGTACGACCTTGAGAGGTAACTCACAAGATTC
   GG
   Site 2 R
```

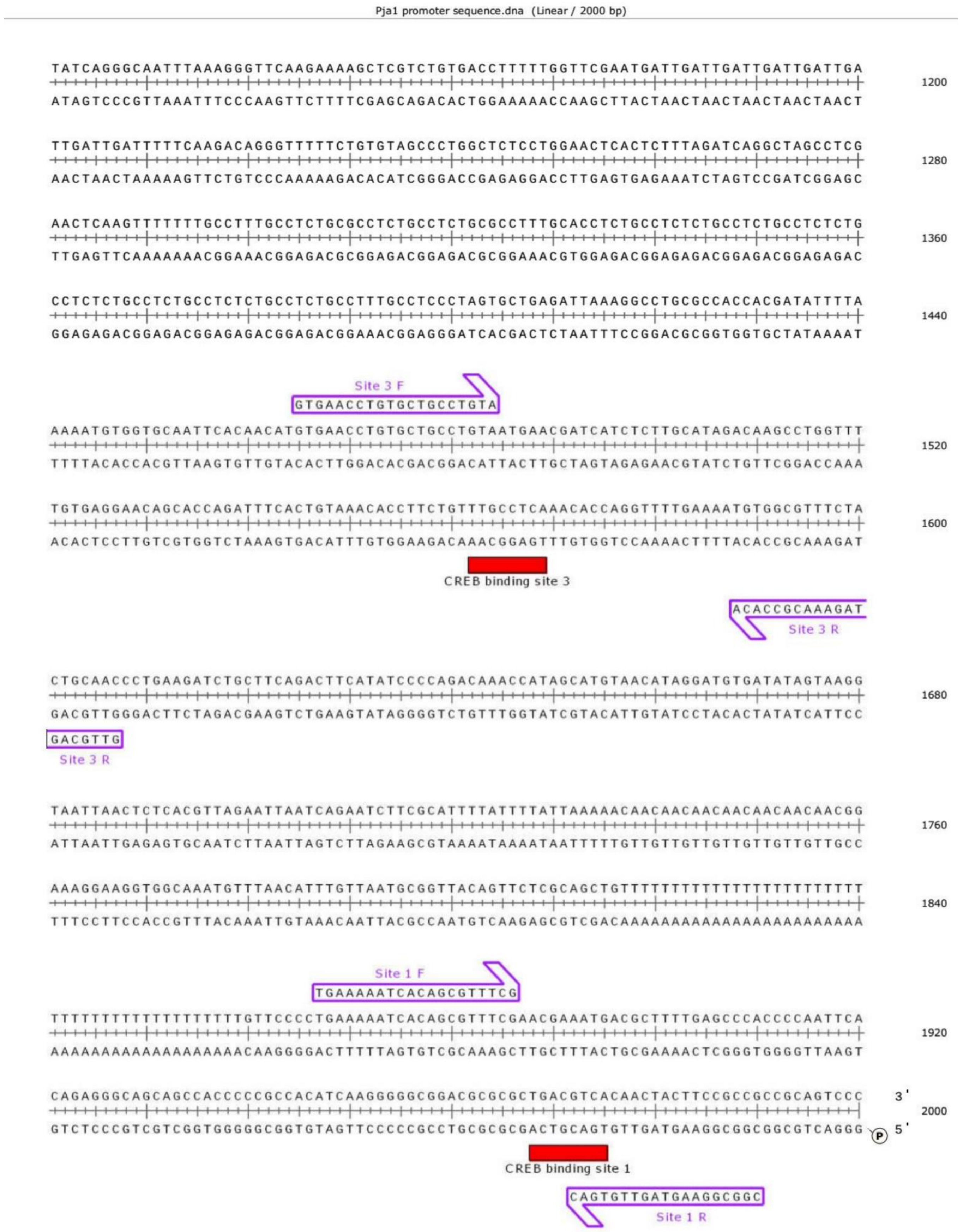

Supplemental Figure 11. Mouse *Pja1* promoter sequence and predicted CREB binding sites

The mouse *Pja1* promoter sequence was obtained from the UCSC database (http://genome.ucsc.edu/). The region 2000 bp upstream and 100 bp downstream of the transcriptional start site was considered the promoter region. Here, we show the 2000 bp upstream of the transcriptional start site. The red rectangles indicate the potential binding sites of CREB. The hollow half arrows indicate the location of the designed primers.

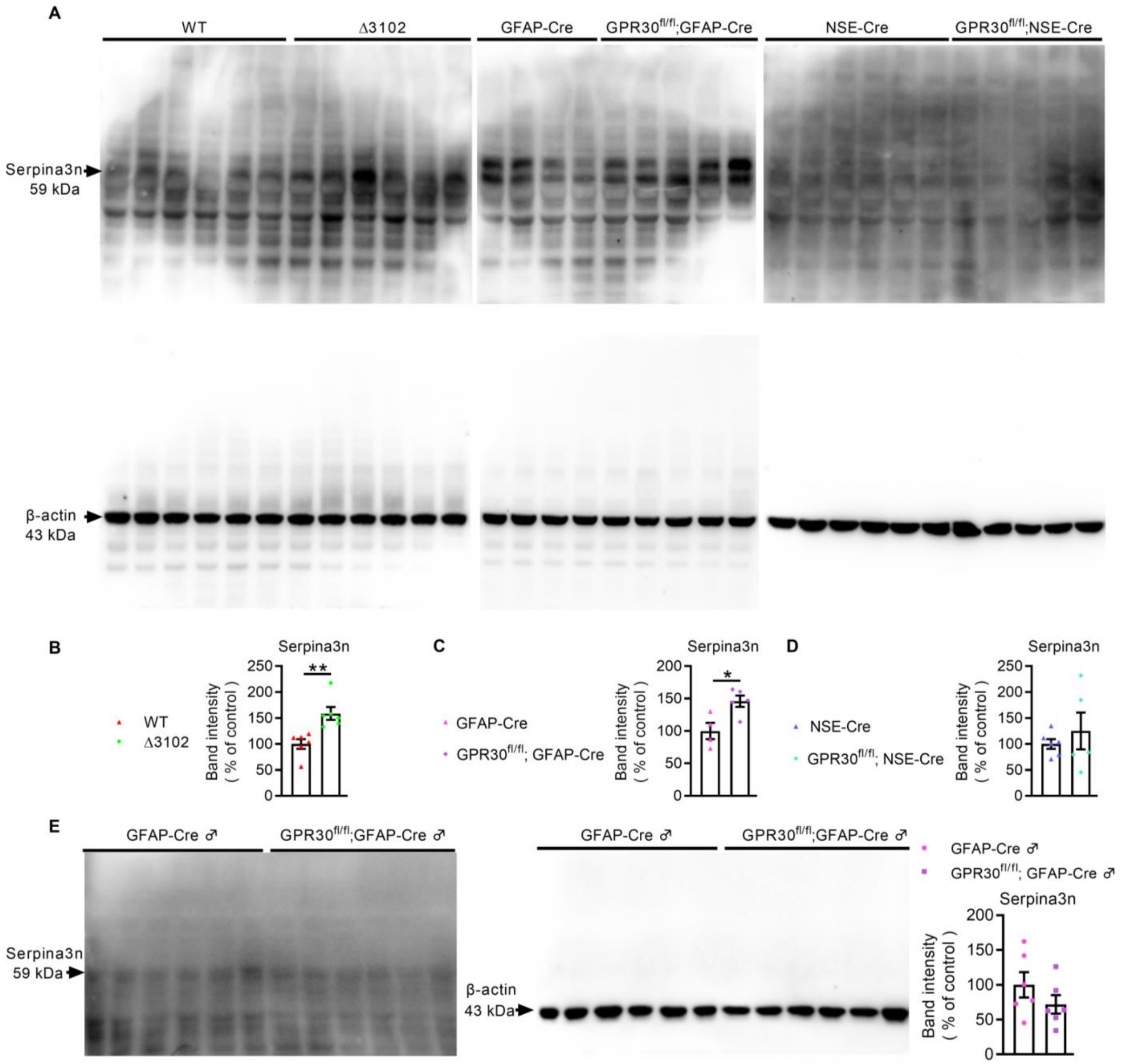

Supplemental Figure 12. The levels of Serpina3n in the hippocampi of female and male transgenic mice

(A-D) Immunoblots of Serpina3n in the hippocampi of Δ3102, AG-KO, and NG-KO female mice. Serpina3n levels were increased in Δ3102 (B, *n* = 6 mice per group) and AG-KO mice (C, *n* = 4 control mice; *n* = 5 AG-KO mice) but unchanged in NG-KO mice (D, *n* = 6 control mice; *n* = 5 NG-KO mice). Δ3102: GRR30 KO. AG-KO: $GPR30^{fl/fl}$;GFAP-Cre. NG-KO: $GPR30^{fl/fl}$;NSE-Cre. WT, GFAP-Cre, and NSE-Cre are control mice. (E) Serpina3n was unchanged in the hippocampi of AG-KO male mice. *n* = 6 mice per group. Data are presented as mean ± SEM. *$P < 0.05$, **$P < 0.01$ by independent sample t test.

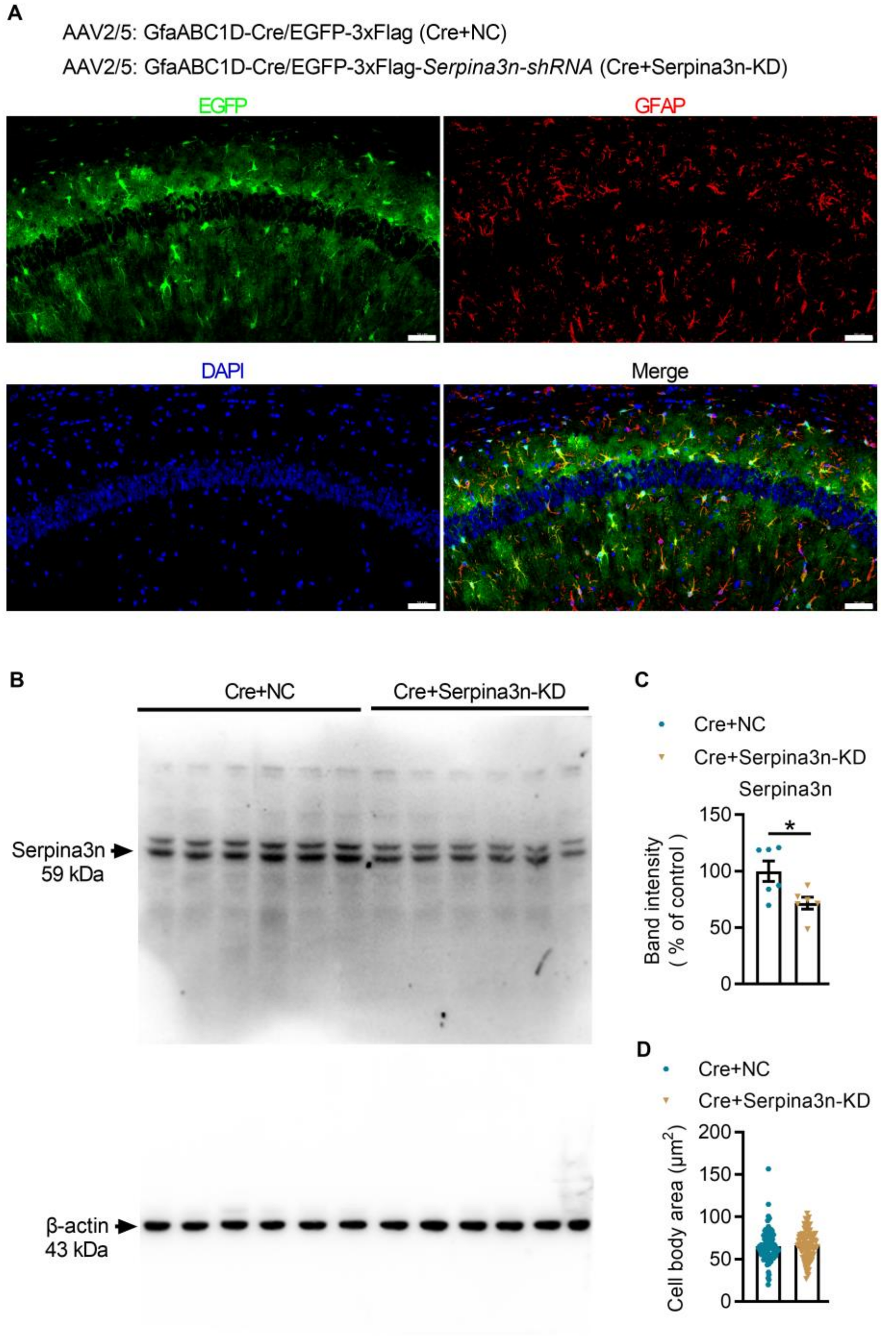

Supplemental Figure 13. Specific Serpina3n knockdown in astrocytes and quantification of the astrocytic cell body area

(A) Images showing the specific expression of viruses in astrocytes of the CA1 area. Scale bar: 50 μm. (B and C) *Serpina3n-shRNA* successfully downregulated Serpina3n in the hippocampi of Cre+Serpina3n-KD mice. n = 6 mice per group. (D) Serpina3n knockdown had no effect on the astrocytic cell body area. n = 100 cells from 3 mice per group. Cre+NC indicates astrocytic GPR30 KO mice. Cre+Serpina3n-KD indicates astrocytic GPR30 KO and Serpina3n knockdown mice. Data are presented as mean ± SEM. $^*P$ < 0.05 by independent sample t test.

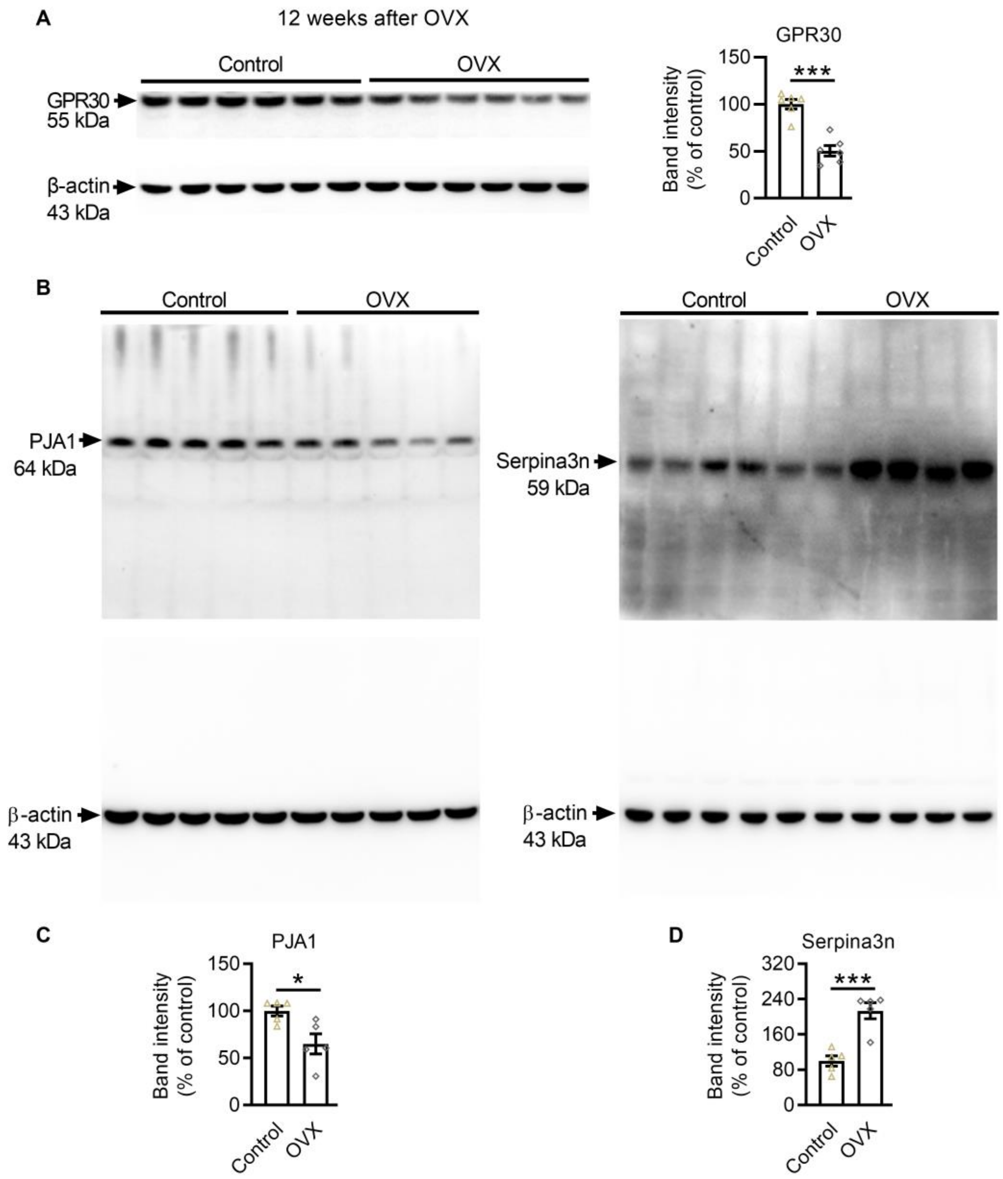

Supplemental Figure 14. GPR30/PJA1/Serpina3n expression in long-term OVX mice

(A) The level of GPR30 was decreased in the hippocampus 12 weeks after ovariectomy. n = 6 mice per group. (B-D) Immunoblots of PJA1 and Serpina3n in hippocampal protein lysates from control mice and OVX mice at 12 weeks after ovariectomy (B). The levels of PJA1 were decreased (C), but the level of Serpina3n was increased in OVX mice (D). n = 5 mice per group. Data are presented as mean ± SEM. *$P < 0.05$, ***$P < 0.001$ by independent sample t test.

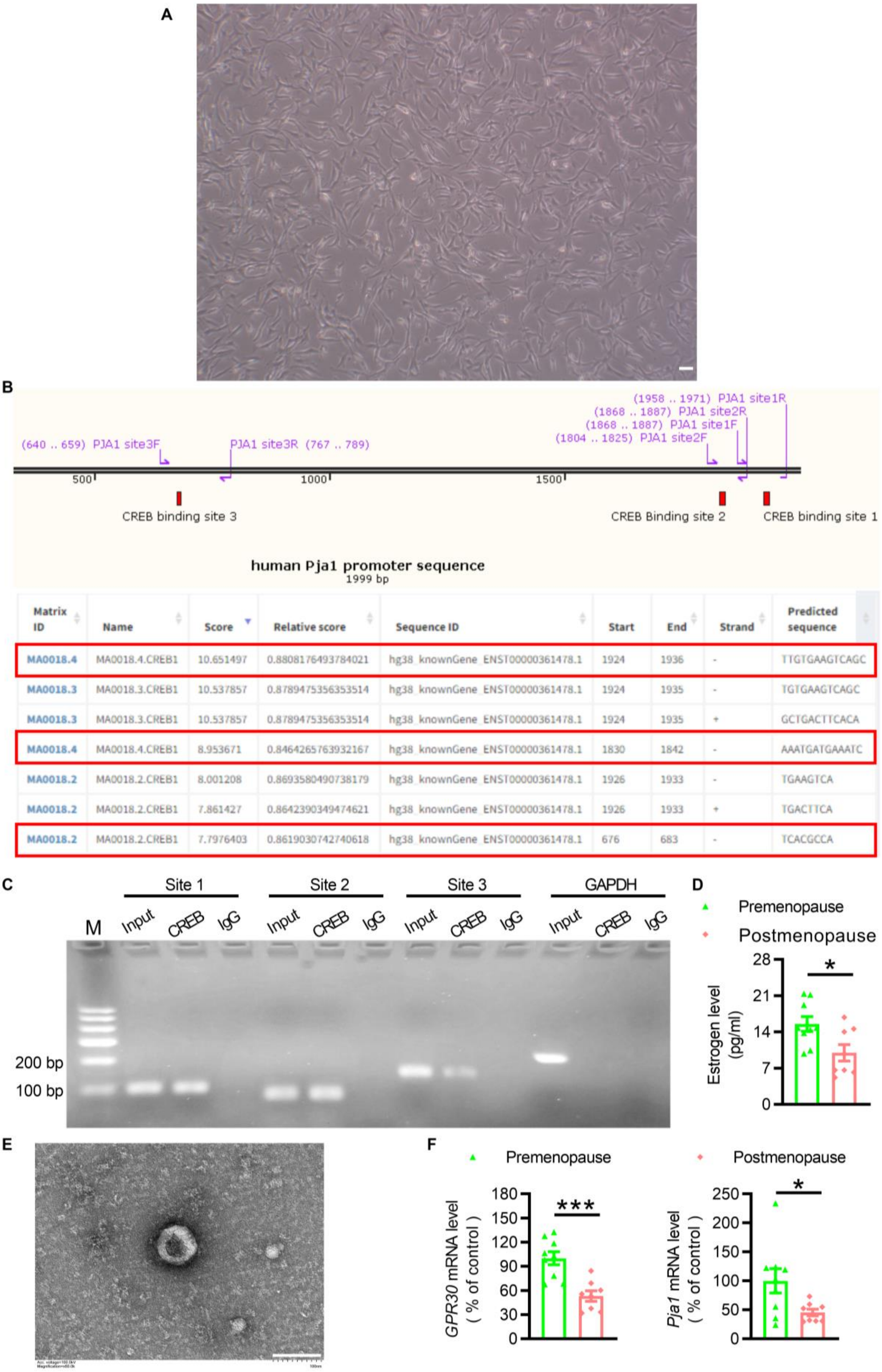

Matrix ID	Name	Score	Relative score	Sequence ID	Start	End	Strand	Predicted sequence
MA0018.4	MA0018.4.CREB1	10.651497	0.8808176493784021	hg38_knownGene_ENST00000361478.1	1924	1936	-	TTGTGAAGTCAGC
MA0018.3	MA0018.3.CREB1	10.537857	0.8789475356353514	hg38_knownGene_ENST00000361478.1	1924	1935	-	TGTGAAGTCAGC
MA0018.3	MA0018.3.CREB1	10.537857	0.8789475356353514	hg38_knownGene_ENST00000361478.1	1924	1935	+	GCTGACTTCACA
MA0018.4	MA0018.4.CREB1	8.953671	0.8464265763932167	hg38_knownGene_ENST00000361478.1	1830	1842	-	AAATGATGAAATC
MA0018.2	MA0018.2.CREB1	8.001208	0.8693580490738179	hg38_knownGene_ENST00000361478.1	1926	1933	-	TGAAGTCA
MA0018.2	MA0018.2.CREB1	7.861427	0.8642390349474621	hg38_knownGene_ENST00000361478.1	1926	1933	+	TGACTTCA
MA0018.2	MA0018.2.CREB1	7.7976403	0.8619030742740618	hg38_knownGene_ENST00000361478.1	676	683	-	TCACGCCA

Supplemental Figure 15. CREB binding sites in the human *Pja1* promoter and GPR30/PJA1/Serpina3n expression in exosomes isolated from women's blood

(A) Image of NHAs cultured in a laboratory dish. Scale bar: 100 µm. (B) Bottom: Prediction of CREB binding sites in the human *Pja1* promoter. The red rectangles indicate the top three CREB binding according to the predicted scores. Top: Primers designed to verify the binding of CREB. (C) Image showing the qRT–

PCR products. (D) Estrogen levels in the plasma of premenopausal and postmenopausal women. n = 9 samples in the premenopausal group. n = 8 samples in the postmenopausal group. (E) Transmission electron microscopy image of exosomes. Scale bar: 100 nm. (F) Relative mRNA levels of GPR30 (left) and Pja1 (right) in exosomes collected from the blood of premenopausal and postmenopausal women. n = 9 samples in the premenopausal group. n = 8 samples in the postmenopausal group. NHA indicates normal human astrocyte. Data are presented as mean ± SEM. *$P < 0.05$, ***$P < 0.001$ by independent sample t test.

Sequence: human Pja1 promoter sequence.dna (Linear / 1999 bp)
Features: 3 visible, 3 total
Primers: 6 visible, 6 total

5' GCAACATGCTTTTTTCACTTAATATATTTATATTAGACCAGTACATATATACATATATACTTGCCTTCTTTCTTTCTTTC 80
3' CGTTGTACGAAAAAAGTGAATTATATAAATATAATCTGGTCATGTATATATGTATATATGAACGGAAGAAAGAAAGAAAG

TTTTTTTTTTTTTTTTTTTTGAGACAGGGTCTGACTATTGCCCAGGCTGGATCACAGTGGCGCAATCTTGGCTCACTGCA 160
AAAAAAAAAAAAAAAAAAAACTCTGTCCCAGACTGATAACGGGTCCGACCTAGTGTCACCGCGTTAGAACCGAGTGACGT

GCCTCAACCTCCTGGGCTCAAGTGATTCTCCCACCTCAGCCTCCCAAGTAGCTGGGACTACAGGCACGTACCATTGCGCC 240
CGGAGTTGGAGGACCCGAGTTCACTAAGAGGGTGGAGTCGGAGGGTTCATCGACCCTGATGTCCGTGCATGGTAACGCGG

AGGGAAATTTTTACGTTTTTTTTGTAGAGATGGGGTTTCACCATATTGTCCAGGCTGGTCTCAACTCCTGGGCTCAAGCA 320
TCCCTTTAAAAATGCAAAAAAAACATCTCTACCCCAAAGTGGTATAACAGGTCCGACCAGAGTTGAGGACCCGAGTTCGT

ATCCTCCTGCCTTGGTCTCCCAAAGTGCTGCGATTACAGGTGTCAACCACCATGTCTGTACATGAGCCACCATGCAACAT 400
TAGGAGGACGGAACCAGAGGGTTTCACGACGCTAATGTCCACAGTTGGTGGTACAGACATGTACTCGGTGGTACGTTGTA

GTTTAAATAAATTTAAACAATTTATTTGTTGAGGAGAATTAAAGTTGTTTGCATTATCTTGCTACACTCCTATTCACATA 480
CAAATTTATTTAAATTTGTTAAATAAACAACTCCTCTTAATTTCAACAAACGTAATAGAACGATGTGAGGATAAGTGTAT

TCTTTCTCCATTTGTGTGTCTCAATAGAACACACAATAGAGCAATTGACTAGAAGTAGAATTGCTAGATGAAAATGTATC 560
AGAAAGAGGTAAACACACAGAGTTATCTTGTGTGTTATCTCGTTAACTGATCTTCATCTTAACGATCTACTTTTACATAG

PJA1 site3F
A

TCCATTAAAAAATTTGATAGTTTCAGATTTCTCACCAATGAAGTTCTATTATTGTTATTATTATCATCATCATTTTGAGA 640
AGGTAATTTTTTAAACTATCAAAGTCTAAAGAGTGGTTACTTCAAGATAATAACAATAATAATAGTAGTAGTAAAACTCT

PJA1 site3F
TGGCATCTTGCTTGGTTGT

TGGCATCTTGCTTGGTTGTCCAGGCTGGAGTGCAGTGGCGTGATCTTCACTCACTTCAGCCTCAACCTCCTGGGCTCACA 720
ACCGTAGAACGAACCAACAGGTCCGACCTCACGTCACCGCACTAGAAGTGAGTGAAGTCGGAGTTGGAGGACCCGAGTGT

CREB binding site 3

CAATCCTCCTGCTTCAGCCTCCTAAGTAGTTGGGACTACAGGCACACACCACCAGCTTGTCATATCTTTCTCAGTGAATA 800
GTTAGGAGGACGAAGTCGGAGGATTCATCAACCCTGATGTCCGTGTGTGGTGGTCGAACAGTATAGAAAGAGTCACTTAT

GTGGTGGTCGAACAGTATAGAAA
PJA1 site3R

AAGCAACCTGTTTTGTTCTCTTTAGTTCTTTTTACTTTGAGTTCTCGATGTTTAGGATATTGATATTGTTACGAGTTTGT 880
TTCGTTGGACAAAACAAGAGAAATCAAGAAAAATGAAACTCAAGAGCTACAAATCCTATAACTATAACAATGCTCAAACA

TTCCTCCTTCCTTTCCTTCCTTTTTTTTTTTTTTGCTTCTTTCCTTCCCTCATTCCCTCTCTCCCTTTTTTCTGCTAGTA 960
AAGGAGGAAGGAAAGGAAGGAAAAAAAAAAAAAACGAAGAAAGGAAGGGAGTAAGGGAGAGAGGGAAAAAAGACGATCAT

TTTTACTCTCATATATTTTTTATTCCTATTTCTGCTTTAGGTGAAACTCACATAAATAGAATATAACTTAGCTTTTAAAA 1040
AAAATGAGAGTATATAAAAAATAAGGATAAAGACGAAATCCACTTTGAGTGTATTTATCTTATATTGAATCGAAAATTTT

ACAAAATGCTCAAAATAAGAGTTTGGTTTTTATTAGGAGATGTTAACCCATAATGCATGCTGATATATTTGGATTCCTGC 1120
TGTTTTACGAGTTTTATTCTCAAACCAAAAATAATCCTCTACAATTGGGTATTACGTACGACTATATAAACCTAAGGACG

ATTTTTGTTTTCTTTTTGCATCCTTTTTTTTGTACCCCCCCAGCAACATTTCTTGACCTTTGTTAAGAATGAGTTTTTCT 1200
TAAAAACAAAAGAAAAACGTAGGAAAAAAAACATGGGGGGGTCGTTGTAAAGAACTGGAAACAATTCTTACTCAAAAAGA

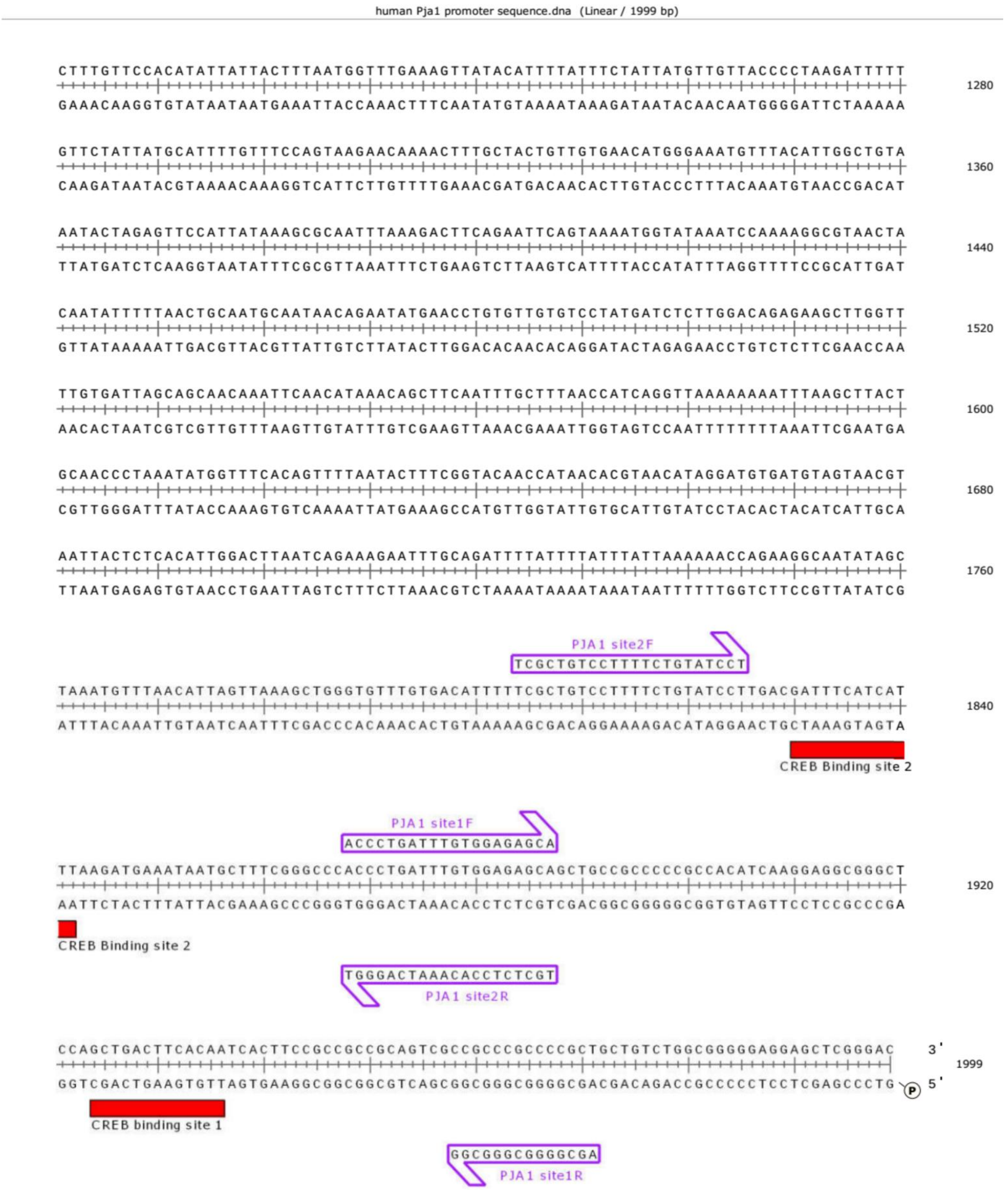

Supplemental Figure 16. Human *Pja1* promoter sequence and predicted CREB binding sites

The promoter sequence of human *Pja1* was obtained from the UCSC database (http://genome.ucsc.edu/). The region 2000 bp upstream and 100 bp downstream of the transcriptional start site was set as the promoter region. Here, we show the middle 1999 bp, which includes the predicted binding sites of CREB. The red rectangles indicate the potential CREB binding sites. The hollow half arrows indicate the location of the designed primers.

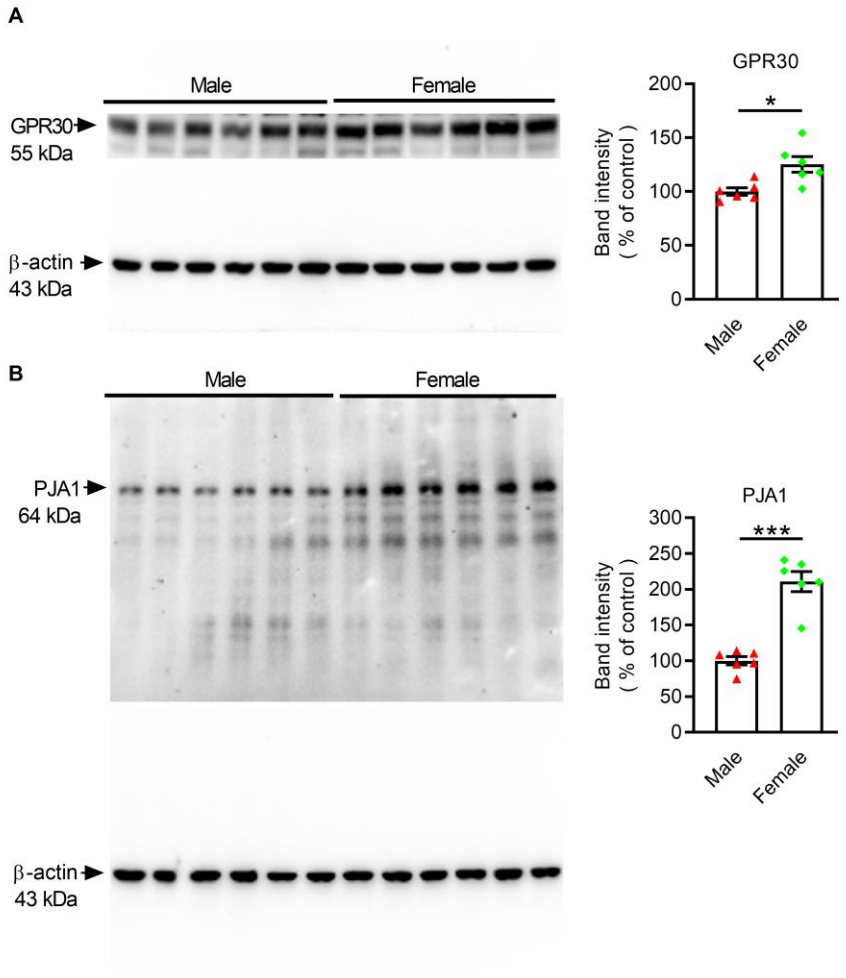

Supplemental Figure 17. Differential levels of GPR30 and PJA1 in the hippocampi of female and male mice

(A and B) The levels of GPR30 (A) and PJA1 (B) were evidently higher in female mice than in male mice. *n* = 6 mice per group. Data are presented as mean ± SEM. **P* < 0.05, ****P* < 0.001 by independent sample t test.

Supplemental Table 1. Sequences of primers used for RT-qPCR

Gene	Sequences (Forward, 5' to 3')	Sequences (Reverse, 5' to 3')
mGAPDH-1	GGTGAAGGTCGGTGTGAACG	CTCGCTCCTGGAAGATGGTG
mGAPDH-2	CTGCTGAAGTGCTCCCTACC	CCCTTTTCTGCCTTCCTACC
mGAPDH-3	TCGTGGAGTCTACTGGTGTCT	GGCGGAGATGATGACCCTTT
hGAPDH	AAAAGCGGGGAGAAAGTAGG	AAGAAGATGCGGCTGACTGT
H2-T23	GGACCGCGAATGACATAGC	GCACCTCAGGGTGACTTCAT
Serping1	ACAGCCCCCTCTGAATTCTT	GGATGCTCTCCAAGTTGCTC
H2-D1	TCCGAGATTGTAAAGCGTGAAGA	ACAGGGCAGTGCAGGGATAG
Ggta1	GTGAACAGCATGAGGGGTTT	GTTTTGTTGCCTCTGGGTGT
ligp1	GGGGCAATAGCTCATTGGTA	ACCTCGAAGACATCCCCTTT
Gbp2	GGGGTCACTGTCTGACCACT	GGGAAACCTGGGATGAGATT
Fbln5	CTTCAGATGCAAGCAACAA	AGGCAGTGTCAGAGGCCTTA
Ugt1a	CCTATGGGTCACTTGCCACT	AAAACCATGTTGGGCATGAT
Fkbp5	TATGCTTATGGCTCGGCTGG	CAGCCTTCCAGGTGGACTTT
Psmb8	CAGTCCTGAAGAGGCCTACG	CACTTTCACCCAACCGTCTT
Srgn	GCAAGGTTATCCTGCTCGGA	TGGGAGGGCCGATGTTATTG
Amigo2	GAGGCGACCATAATGTCGTT	GCATCCAACAGTCCGATTCT
Clcf1	CTTCAATCCTCCTCGACTGG	TACGTCGGAGTTCAGCTGTG
Ptx3	AACAAGCTCTGTTGCCCATT	TCCCAAATGGAACATTGGAT
S100a10	CCTCTGGCTGTGGACAAAAT	CTGCTCACAAGAAGCAGTGG
Sphk1	GATGCATGAGGTGGTGAATG	TGCTCGTACCCAGCATAGTG
Cd109	CACAGTCGGGAGCCCTAAAG	GCAGCGATTTCGATGTCCAC
Ptgs2	GCTGTACAAGCAGTGGCAAA	CCCCAAAGATAGCATCTGGA
Emp1	GAGACACTGGCCAGAAAAGC	TAAAAGGCAAGGGAATGCAC
Slc10a6	GCTTCGGTGGTATGATGCTT	CCACAGGCTTTTCTGGTGAT
Tm4sf1	GCCCAAGCATATTGTGGAGT	AGGGTAGGATGTGGCACAAG
B3gnt5	CGTGGGGCAATGAGAACTAT	CCCAGCTGAACTGAAGAAGG
Cd14	GGACTGATCTCAGCCCTCTG	GCTTCAGCCCAGTGAAAGAC
TNF-α	TGTGCTCAGAGCTTTCAACAA	CTTGATGGTGGTGCATGAGA
IL-1β	TGCCACCTTTTGACAGTGATG	TGATGTGCTGCTGCGAGATT
IL-6	GACTGGGGATGTCTGTAGCTC	CAACTGGATGGAAGTCTCTTGC

mGPR30	TCACCTGGATGAGCTTCGACA	GCTCGCACGATGAGGGAGTA
hGPR30	ATGGATGTGACTTCCCAAGC	GAACAGGCCGATCACGTACT
mPJA1	CCAGCAAGCAAGGAGAGCATT	GCAGCAGATAGGACAGCACATT
hPJA1	GACTTCTGGACGCACAGTGA	CAGTGCTGGCACTCACCTTA
cel-miR-39	AGCCCGTCACCTGGTGTAAATC	CAGTGCAGGGTCCGAGGTAT
mPJA1-site1	TGAAAAATCACAGCGTTTCG	CGGCGGAAGTAGTTGTGAC
mPJA1-site2	GCAGAGCTGGGATCAAAGAC	GGATATCGAACAAGGGGACA
mPJA1-site3	GTGAACCTGTGCTGCCTGTA	GTTGCAGTAGAAACGCCACA
hPJA1-site1	ACCCTGATTTGTGGAGAGCA	AGCGGGGCGGGCGG
hPJA1-site2	TCGCTGTCCTTTTCTGTATCCT	TGCTCTCCACAAATCAGGGT
hPJA1-site3	ATGGCATCTTGCTTGGTTGT	AAAGATATGACAAGCTGGTGGTG
Sry	CGTGGTGAGAGGCACAAGT	TGTGATGGCATGTGGGTTCC

Supplemental methods

Fiber photometry recording. A two-color multichannel optical fiber recording system (410&470) (RWD Life Technology) was used to assess the dynamics of intracellular Ca^{2+} concentrations in astrocytes. Mice were injected with AAV2/5-GfaABC1D-GCaMP6f (1 µl) into the CA1 region of the hippocampus. An optical fiber (diameter of 200 µm) was implanted in the virus injection site. The mice were allowed to recover for 4 weeks before FC training. The light intensity at the tip of the fiber was 0.03 mW (470 nm) or 0.015 mW (410 nm). Astrocytic Ca^{2+} signals and behavior were recorded simultaneously. ΔF/F signals over the entire training session were calculated to reflect astrocyte activity.

Immunostaining after FISH. Brain sections were obtained as described above. FISH was performed by using an RNASweAMI™ FISH kit (GF010-50T, Servicebio) according to the manufacturer's instructions. Briefly, brain sections were incubated with fixative solution, followed by proteinase K (20ug/ml) digestion. After washing, the sections were incubated with pre-hybridization solution, GPR30 probes (synthesized by Servicebio), branch probes, and FITC fluorescent probes in turn. After FISH, the sections were immunostained with GFAP (GB11096, Servicebio) or NeuN (GB11138, Servicebio). Finally, fluorescence images were acquired using a confocal laser-scanning microscope (Nikon ECLIPSE C1).

Isolation, characterization, and mRNA expression analysis of exosomes from women's blood. Blood from premenopausal (aged 35-45 years, normal menstrual cycles) and postmenopausal (aged 50-65 years, amenorrhea for more than 1.5 years) women without underlying diseases and hormone therapy was collected. Blood samples (10 ml) were collected in a K_2EDTA-containing tube and immediately centrifuged at 2,000 × g for 10 min at 4°C. Plasma was used for exosome purification by Allwegene Technologies. Briefly, plasma samples were subjected to ultracentrifugation at 10,000 × g for 45 min at 4 °C to remove large vesicles, passed through 0.45 µm filters, and spun at 100,000 × g for 70 min at 4 °C. The exosomes were washed once with precooled 1× PBS and resuspended for further characterization. A total of 10 µl of exosomes were adsorbed on copper meshes and fixed/stained by adding 10 µl of uranyl acetate. The meshes

were dried for several minutes at room temperature and finally examined using an HT-7700 transmission electron microscope (Hitachi).

Total RNA was extracted from the isolated exosomes using a miRNeasy Mini Kit (#217004, Qiagen) according to the manufacturer's instructions. Reverse transcription (RT) was performed using a RevertAid First Strand cDNA Synthesis Kit (with K1622, Thermo). Cel-miR-39 was added to the RT systems at a constant proportion as an external reference. The cDNAs were used for qRT–PCR using SYBR Green qPCR Master Mix (4943914001-SR, Roche), and mRNA levels are expressed relative to the level of cel-miR-39. The primer sequences are listed in Supplemental Table 1.
